# Carbonaceous Components Underlying Wildfire Smoke PM_2.5_-Associated Mortality in Older U.S. Adults

**DOI:** 10.1021/acs.est.6c07219

**Published:** 2026-09-30

**Authors:** Zhihao Jin, Ke Xu, Hua Hao, Meng Qi, Gonzalo A. Ferrada, Joshua S. Fu, Noah Scovronick, Howard H. Chang, Alvaro Alonso, Kyle Steenland, Yang Liu

**Affiliations:** † Gangarosa Department of Environmental Health, Rollins School of Public Health, 1371 Emory University, Atlanta, Georgia 30322, United States; ‡ Department of Civil and Environmental Engineering, 4292 University of Tennessee, Knoxville, Tennessee 37996, United States; § Integrated Computational Earth Sciences Group, Computational Sciences and Engineering Division, Oak Ridge National Laboratory, Oak Ridge, Tennessee 37831, United States; ∥ Department of Biostatistics and Bioinformatics, Rollins School of Public Health, Emory University, Atlanta, Georgia 30322, United States; ⊥ Department of Epidemiology, Rollins School of Public Health, Emory University, Atlanta, Georgia 30322, United States

**Keywords:** wildland fire, mortality, fine particulate matter, carbonaceous PM_2.5_, medicare

## Abstract

Wildland fire activity has increased in the U.S., elevating population exposure to smoke-derived fine particulate matter (PM_2.5_). Emerging studies suggest higher toxicity per unit mass for PM_2.5_ from wildfire smoke. However, source-resolved evidence on long-term mortality effects of major carbonaceous components in smoke PM_2.5_ remains scarce. We linked annual, high-resolution estimates of smoke and non-smoke PM_2.5_ mass and carbonaceous matter to 77,470,752 Medicare beneficiaries during 2002–2019 and fit stratified Cox models. Long-term exposure to smoke PM_2.5_ mass and its carbonaceous fraction were associated with higher all-cause mortality risk per unit mass than their non-smoke counterparts. Per 1 μg/m^3^ increase, hazard ratios were 1.032 (95% CI: 1.027–1.037) for smoke PM_2.5_ mass and 1.087 (1.076–1.098) for smoke carbonaceous components. On the interquartile-range scale, the association was larger for non-smoke PM_2.5_ than for smoke PM_2.5_, whereas smoke carbon remained more strongly associated with mortality. Associations were stronger among Black and other racial groups and Medicaid-eligible beneficiaries. Substantial spatial heterogeneity was observed across empirical smoke regimes and geographic regions. The associations correspond to 22,420 annual deaths attributable to smoke PM_2.5_, with the carbonaceous fraction accounting for most of this burden. These findings support strengthening wildfire smoke health-impact assessment and mitigation as climate change increases population exposure to wildfire smoke.

## Graphical abstract

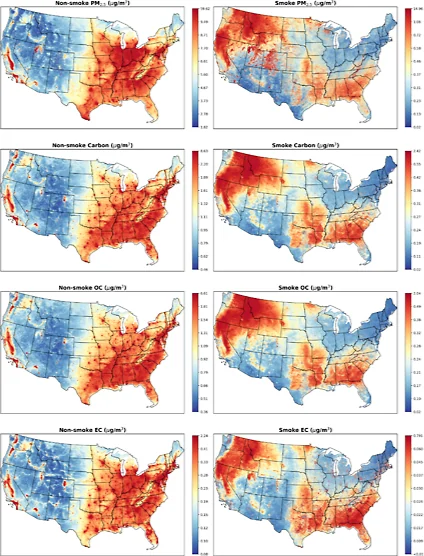

## Introduction

Wildland fire activity in the U.S. has intensified over recent decades, driven by anthropogenic warming, expanding fuel loads, and lengthening fire seasons.^1,2^ Climate-driven increases in wildfire smoke could become the dominant source of climate-related health impacts in the U.S., with economic damages that may exceed those from all other climate impacts combined.^3^ Wildfire smoke is a complex mixture of air pollutants and contains substantial PM_2.5_.^4^ PM_2.5_ has demonstrated adverse health impacts because it can penetrate the respiratory system through inhalation and trigger oxidative stress, systemic inflammation, and cardiopulmonary dysfunction.^5,6^ Emerging long-term epidemiological studies that distinguish smoke from non-smoke PM_2.5_ suggest that the smoke-derived fraction has higher toxicity per unit mass. In a U.S. Medicare cohort, Xu et al. reported that each 1 μg/m^3^ increase in smoke PM_2.5_ was associated with a 9.2% increase in chronic obstructive pulmonary disease mortality, whereas the corresponding increase for non-smoke PM_2.5_ was 1.0%.^7^ Similarly, Hao et al. found that long-term exposure to fire smoke PM_2.5_ conferred a higher risk of incident heart failure (Hazard ratio [HR] per 1 μg/m^3^: 1.014) than non-smoke PM_2.5_ (HR: 1.005).^8^ National burden analyses in the U.S. further suggested that long-term exposure to wildfire smoke PM_2.5_ contributed about 11,000–24,000 deaths per year.^9,10^ However, it remains unclear which chemical constituents are responsible for its elevated toxicity.

A biologically plausible driver of the elevated toxicity of smoke PM_2.5_ is the carbonaceous composition of smoke. The health effects of PM_2.5_ may vary according to its chemical composition including carbonaceous matter, secondary inorganic aerosols, metals, and mineral particles. Although these constituents have each been associated with adverse health outcomes, the relative magnitudes of these associations vary across source mixtures, populations, and regions.^11,12^ Among these PM_2.5_ constituents, organic carbon (OC) and elemental carbon (EC) are particularly relevant to wildfire smoke because they are major products of biomass burning and incomplete combustion.^13,14^ Carbonaceous particles may influence health through several complementary pathways, including reactive oxygen species formation, oxidative stress, pulmonary and systemic inflammation, and cardiovascular dysfunction.^15,16^ Yet, OC and EC also originate from nonsmoke sources, including fossil-fuel combustion, traffic, and industry. Consequently, smoke and nonsmoke carbonaceous particles may differ in their associated chemical mixtures and physicochemical characteristics and may not be toxicologically equivalent per unit mass.^17,18^ Epidemiological studies of PM_2.5_ speciation frequently implicate carbonaceous species in mortality, but source-resolved evidence regarding the long-term mortality effects of smoke-specific carbonaceous components remains limited.^11,19^ This gap reflects the lack of long-term, high-resolution exposure estimates that distinguish smoke from nonsmoke sources.

Smoke composition and toxicity may also vary geographically according to fuel types, combustion conditions, plume transport, and atmospheric processes.^20^ Smoke generated in different regions can differ in primary emissions, plume injection heights, transport distances, and degrees of photochemical aging, which may alter particle size distributions, oxidative potentials, and biological activities.^13,21^ However, most epidemiological studies have implicitly assumed uniform toxicity of smoke PM_2.5_ across space without evaluating whether health risks differ across distinct smoke regimes. In addition, associations may differ across demographic and socioeconomic groups because of variations in underlying susceptibility, exposure patterns, and access to protective resources.

To address these gaps, we linked national, high-resolution estimates of smoke-specific PM_2.5_ mass and total carbonaceous components (carbon, i.e., OC + EC) to older U.S. Medicare beneficiaries. The primary objectives of this study were to quantify associations of long-term exposure to smoke PM_2.5_ and smoke carbon with all-cause mortality, compare these associations with those for their nonsmoke counterparts, and evaluate whether carbonaceous constituents are associated with the elevated mortality risk observed for smoke PM_2.5_. Secondary objectives were to assess heterogeneity across population groups, smoke regimes, and U.S. geographic regions and estimate the mortality burden attributable to smoke PM_2.5_ and smoke carbon.

## Materials and Methods

### Data and Study Population

Medicare is a federal health insurance program that covers nearly all U.S. adults who are aged 65 years or older. The program comprises two components: traditional fee-for-service Medicare, in which the federal government pays providers directly, and Medicare Advantage, in which private insurers receive capitated payments to administer benefits and manage care. The claims and enrollment data used in this study include beneficiaries enrolled in either of these components. We constructed an open cohort from the Centers for Medicare and Medicaid Services (CMS) denominator file, which is a privacy-protected national registry of Medicare enrollment and demographics. We included beneficiaries aged 65–100 years residing in the contiguous United States (CONUS) during 2002–2019. Individuals contributed person-time in eligible years and were followed until death, loss to follow-up, or the end of 2019. For each beneficiary and calendar year, we extracted demographic variables (i.e., sex, age, race, residential ZIP code), Medicaid eligibility, and Medicare Advantage status as indicators of socioeconomic status (SES) and the recorded date of death (up to December 31, 2019). This study was approved by the Emory University Institutional Review Board (approval number STUDY00004823) and conducted under a data use agreement with the Centers for Medicare & Medicaid Services. Informed consent was waived because the study involved a secondary analysis of existing administrative data.

### Estimation of Wildfire Smoke PM_2.5_ and Its Carbonaceous Fraction

We used previously developed national predictions of daily 1 km concentrations of PM_2.5_ mass, OC, and EC separately for smoke and nonsmoke sources for 2002–2019 across the CONUS. Details of the modeling framework have been published elsewhere.^22^ In brief, we developed a three-level residual-adjusted superlearner framework based on machine learning algorithms, combining ground observations, chemical transport model simulations of the Community Multiscale Air Quality Modeling System (CMAQ), satellite-based aerosol optical depth products, and multiple auxiliary predictors. The exposure models were developed for nonsmoke and fire smoke-impacted scenarios and were calibrated directly against ground-based measurements from different national monitoring networks (3081 and 410 monitoring stations for PM_2.5_ and OC/EC, respectively), ensuring the models were anchored to observed data. Smoke-specific concentrations were then derived as the difference between smoke-impacted and nonsmoke predictions. Ten-fold cross-validation versus ground-based observations at the annual scale showed high prediction accuracy: OC coefficients of determination (*R* ^2^) = 0.979–0.980, EC *R* ^2^ = 0.980–0.985, and PM_2.5_ mass *R* ^2^ = 0.901–0.939 (Table S1).

We averaged daily 1 km predictions to annual ZIP-code means and assigned exposures by beneficiaries’ annually updated residential ZIP codes, assuming homogeneous exposure within ZIP codes. To better reflect population exposure within ZIP codes, we calculated population-weighted annual mean concentrations, using gridded annual population data from the LandScan database to weight 1 km predictions before aggregation.^23^ We defined PM_2.5_ carbonaceous components (carbon) as the sum of OC and EC. This metric was selected as the primary carbonaceous exposure because separate OC and EC estimates may be sensitive to collinearity (Table S2) and shared measurement uncertainty. The combined metric was not intended to imply that OC and EC are biologically equivalent. Long-term exposure was defined as the 3 year moving average of the prior calendar years. For example, the exposure for 2010 is the average concentration in 2007–2009. To ensure complete exposure histories, we excluded person-years in 2002–2004, so the analytic follow-up began January 1, 2005. Descriptive statistics for the resident population, number of Medicare beneficiaries, and land area of the ZIP codes used for exposure assignment are provided in Table S3.

### Covariates

Models accounted for individual-level characteristics by stratifying the baseline hazard by sex, race, Medicaid insurance status, and 5 year age-at-entry group and by including attained age and Medicare Advantage status as annually updated time-varying covariates. Models also adjusted for time-varying neighborhood covariates, including ZIP-code socioeconomic status (SES) indicators from the U.S. Census Bureau/American Community Survey (i.e., percentage of Black population, percentage of people living in rental housing or apartments, percentage of population living below the poverty line, percent of population aged 65 or above, and total population), ZIP-code-level meteorological factors from the North American Land Data Assimilation System (i.e., 3 year moving average relative humidity and temperature), healthcare access (i.e., distance to the nearest hospital) from the American Hospital Association Survey, and the behavioral factor (i.e., county-level smoking prevalence and mean body mass index [BMI]) from the Behavioral Risk Factor Surveillance System. The meteorological averaging period corresponded to the exposure assessment window and was intended to account for long-term spatial and temporal meteorological variation. To further account for broad-scale spatial heterogeneity in wildfire smoke PM_2.5_ composition, frequency, and transport conditions, we adjusted for a ZIP-code-level categorical smoke regime variable (Figure S1), consistent with evidence of substantial spatial heterogeneity in combustion-related OC and EC.^21,24^ To address residual temporal and spatial heterogeneity, we included indicator variables for calendar year and five U.S. geographic regions (i.e., Northeast, Southeast, Midwest, West, Southwest [Figure S2]). This classification was adapted from a commonly used geographic grouping of the United States based on the relative locations of states within the country.^8,25,26^ Unless otherwise noted, covariates entered the models as linear, time-varying terms. Details of the smoke regime definition and interpretation are provided in Section S1.

### Statistical Analysis

We estimated the associations of long-term exposure to smoke PM_2.5_ mass and smoke carbon with all-cause mortality using time-varying Cox proportional hazards models with years since cohort entry as the underlying time scale. Baseline hazards were stratified by age at study entry (5 year groups), sex (male and female), race (Black, White, and Others), and Medicaid eligibility to account for non-proportional hazards across these strata. For each pollutant, we included both smoke and non-smoke concentration terms for a given pollutant in the same model so that the effect estimates for smoke and non-smoke are mutually adjusted. Robust standard errors were adopted to account for residual within-ZIP correlation. Primary effect estimates are reported as hazard ratios (HRs) and 95% confidence intervals (CIs) per 1 μg/m^3^ and per interquartile range (IQR, 25th–75th percentiles) increase in exposure. Because long-term smoke PM_2.5_ concentrations are typically below 1 μg/m^3^, we also reported HRs per 0.1 μg/m^3^ and 90% inter-percentile range (90IPR, 5th–95th) increments with 95% CIs, to represent more realistic and extreme exposure contrasts.

We further evaluated potential nonlinear exposure–response relationships using penalized regression splines. Additionally, to estimate the effect modification by sex, race, and SES status, we conducted subgroup analyses via stratified fits. These effect modifiers were selected based on their role in shaping susceptibility to PM_2.5_-related mortality, as documented in previous studies.^8,27^ Effect modification was evaluated by including multiplicative interaction terms between smoke exposure and each potential effect modifier in the fully adjusted model. Statistical heterogeneity was assessed using Wald χ-square tests. For effect modifiers with more than two categories, all of the interaction terms were tested jointly. Two-sided interaction *p*-values are reported. To explore spatial heterogeneity in associations caused by different fire smoke sources and compositions, climatic conditions, and population characteristics, we compared models fitted within the smoke regimes and U.S. geographic regions. We also performed sensitivity analyses to evaluate the robustness of our main results, including (1) using 1-, 2-, and 5 year moving averages of smoke PM_2.5_, (2) restricting analysis to nonmovers and further restricting nonmovers to ZIP codes with low within-ZIP variability of smoke to reduce potential exposure misclassification, (3) excluding the temperature and relative humidity covariates, (4) combining smoke regimes 1 and 2 and repeated the primary models using the resulting four-regime classification, (5) separating analyses for OC, EC, and noncarbonaceous PM_2.5_ (i.e., total mass minus carbon), (6) fitting two additional models, one including smoke OC and smoke EC simultaneously and the other including smoke OC, smoke EC, and smoke noncarbonaceous PM_2.5_ simultaneously, (7) using unweighted exposure to enable comparison with prior studies, and (8) adjusting for claims-based indicators of tobacco use disorder, alcohol use disorder, and drug use disorder obtained from the CCW Other Chronic or Potentially Disabling Conditions segment. Additional details regarding the implementation of these sensitivity analyses are provided in Section S2.

### Attributable Mortality Burden

We estimated the mortality burden attributable to smoke and non-smoke PM_2.5_ among U.S. adults aged ≥65 years for each county and year. Population-attributable fractions (PAFs) were derived from primary effect estimates and annual county-level exposure, which were then applied to county-year all-cause death counts (aged ≥65, 2005–2019) obtained from the CDC WONDER dataset. Because separate component PAFs are not additive, we partitioned the total PM_2.5_-related burden into smoke and non-smoke PM_2.5_ and then allocated the smoke burden to PM_2.5_ carbon and the non-carbonaceous fraction using a Shapley attribution (details in Section S3).

## Results

### Cohort Characteristics and Exposure Patterns

The study cohort included nearly 77 million Medicare beneficiaries from 2005 to 2019, with a total of approximately 640 million person-years (median follow-up: 8 years) and 27,687,790 death records. Participants were 54.80% female, 82.56% identified as White, and 19.07% were Medicaid-eligible (Table 1). The mean 3 year moving average concentrations were 0.421 μg/m^3^ for smoke PM_2.5_ mass and 0.295 μg/m^3^ for smoke carbon, with corresponding IQRs of 0.305 and 0.247 μg/m^3^, respectively. The average concentrations of non-smoke PM_2.5_ and carbon were 9.171 and 3.019 μg/m^3^, with corresponding IQRs of 3.038 and 0.936 μg/m^3^, respectively. Smoke PM_2.5_ and carbon were highest in the Southeast and West, whereas non-smoke concentrations were higher across densely populated areas of the Eastern U.S. (Figure 1). Long-term OC-to-EC ratios also exhibited substantial spatial heterogeneity for both smoke and non-smoke sources (Figure S3). Detailed exposure distributions are summarized in Table S4. The five smoke regimes exhibited distinct and interpretable differences (Table S6): regimes 1–2 reflected relatively fresh, near-source smoke, or short-range transported smoke, whereas regimes 3–5 reflected greater influence of transported smoke, fewer heavy-smoke days, and higher smoke carbon contributions. Detailed characteristics of smoke regimes are documented in Section S1 and Tables S5 and S6.

**Table 1:** Descriptive Statistics for the Study Population and Distributions of 3 Year Moving Average Exposure Levels

variable	number	percentage
total population	77,470,752	100.00%
death	27,687,790	35.74%
total person-years	637,638,834	
median follow-up years	8	
Sex		
male	35,012,998	45.20%
female	42,457,754	54.80%
Race		
White	63,962,060	82.56%
Black	7,185,568	9.28%
Other^a^	6,323,124	8.16%
Entry Age		
65–69	50,282,455	64.91%
70–74	8,698,174	11.23%
75–79	7,459,831	9.63%
80–84	5,779,819	7.46%
85+	5,250,473	6.78%
Medicaid Eligibility		
dual-eligible	14,776,230	19.07%
non-dual eligible	62,694,522	80.93%
U.S. Geographic Region^b^		
Midwest	17,250,346	22.27%
Northeast	14,700,516	18.98%
Southeast	22,949,313	29.62%
Southwest	8,381,881	10.82%
West	14,188,696	18.31%
Smoke Regime		
1	775,151	1.00%
2	5,385,545	6.95%
3	38,726,513	49.99%
4	25,005,841	32.28%
5	7,577,702	9.78%

3 year exposure^c^ (μg/m^3^)	mean ± SD	IQR/90IPR
smoke PM_2.5_ mass	0.421 ± 0.371	0.305/0.912
smoke PM_2.5_ carbon	0.295 ± 0.209	0.247/0.644
smoke PM_2.5_ OC	0.246 ± 0.174	0.207/0.535
smoke PM_2.5_ EC	0.049 ± 0.039	0.044/0.119
non-smoke PM_2.5_ mass	9.171 ± 2.269	3.038/7.476
non-smoke PM_2.5_ carbon	3.019 ± 0.902	0.936/2.620
non-smoke PM_2.5_ OC	2.291 ± 0.639	0.585/1.838
non-smoke PM_2.5_ EC	0.728 ± 0.300	0.375/0.962

a Other indicates Asian, Hispanic, Native American, Pacific Islander, and multiracial individuals.

b Regions were obtained on the basis of the first follow-up year.

c Exposure was population-weighted on the basis of the LandScan global population database.

**Figure 1:**
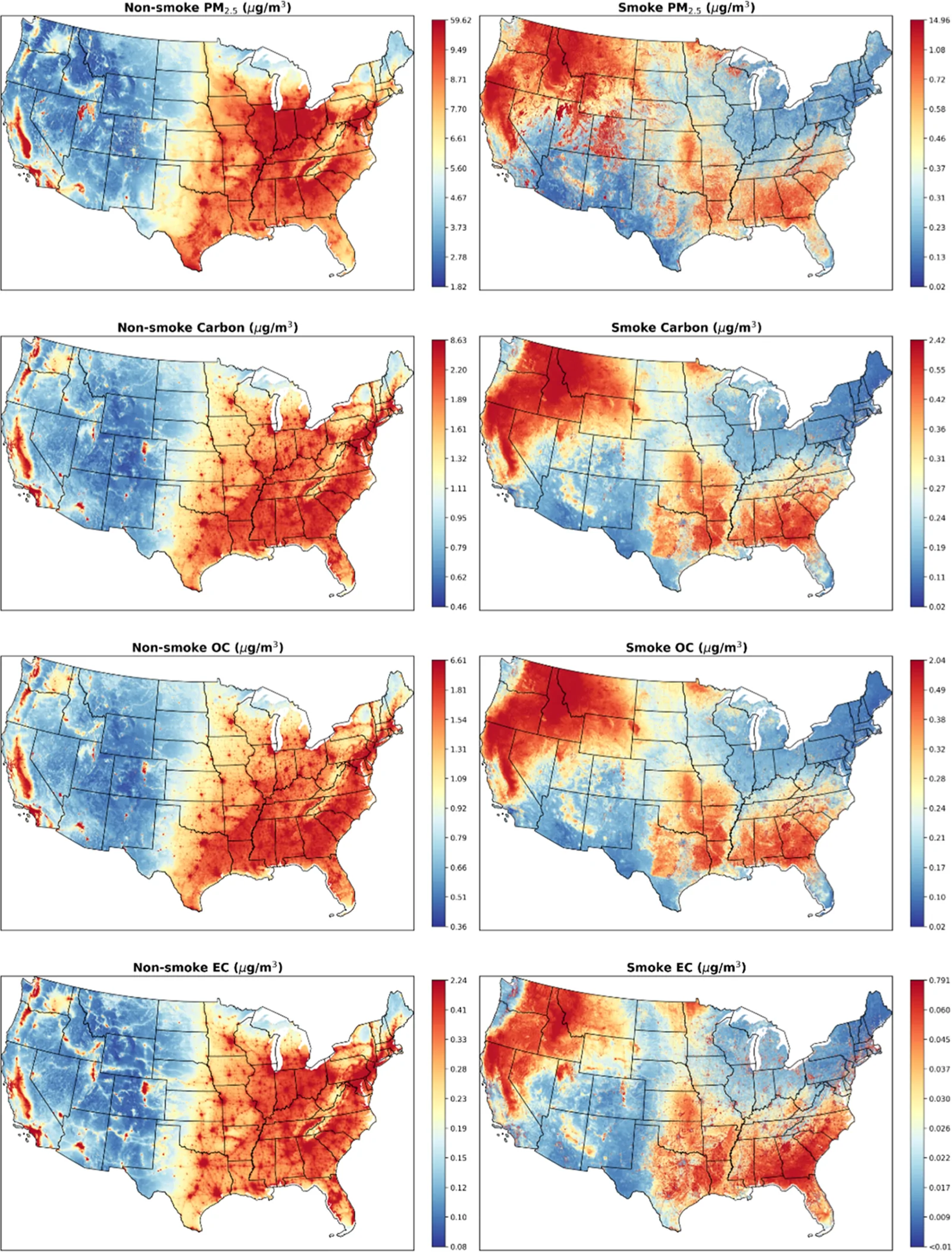
Mean annual concentrations (μg/m^3^) of non-smoke (left) and smoke (right) PM_2.5_ mass, carbon (OC + EC), OC, and EC across the CONUS, 2002–2019. Mapped with 1 km spatial resolution.

### Effect Estimates

In fully adjusted models, higher long-term exposure to smoke PM_2.5_ mass and smoke carbon were each significantly associated with higher all-cause mortality risk and generally showed larger effects than their non-smoke counterparts (Figure 2 and Table S7). On a per 1 μg/m^3^ basis, smoke PM_2.5_ (HR: 1.032 [95% CI: 1.027–1.037]) and carbon (1.087 [1.076–1.098]) each showed stronger associations with mortality than their non-smoke counterparts (PM_2.5_: 1.009 [1.008–1.011]; carbon: 1.013 [1.010–1.017]). Using IQR contrasts, the association for non-smoke PM_2.5_ (1.029 [1.025–1.033]) was greater than that for smoke PM_2.5_ (1.010 [1.008–1.011]), while smoke carbon (1.021 [1.018–1.023]) showed larger effect sizes than the non-smoke carbon (1.012 [1.009–1.016]). Under a 0.1 μg/m^3^ increase in long-term smoke PM_2.5_ mass and carbon, applied to the 2023 U.S. deaths among adults aged ≥65 (2,310,872), the population attributable fractions were 0.32% (95% CI: 0.27%–0.37%) and 0.83% (0.73%–0.93%), respectively. These corresponded to approximately 7395 (6239–8550) and 19,180 (16,869–21,491) additional annual deaths among U.S. older adults.

**Figure 2:**
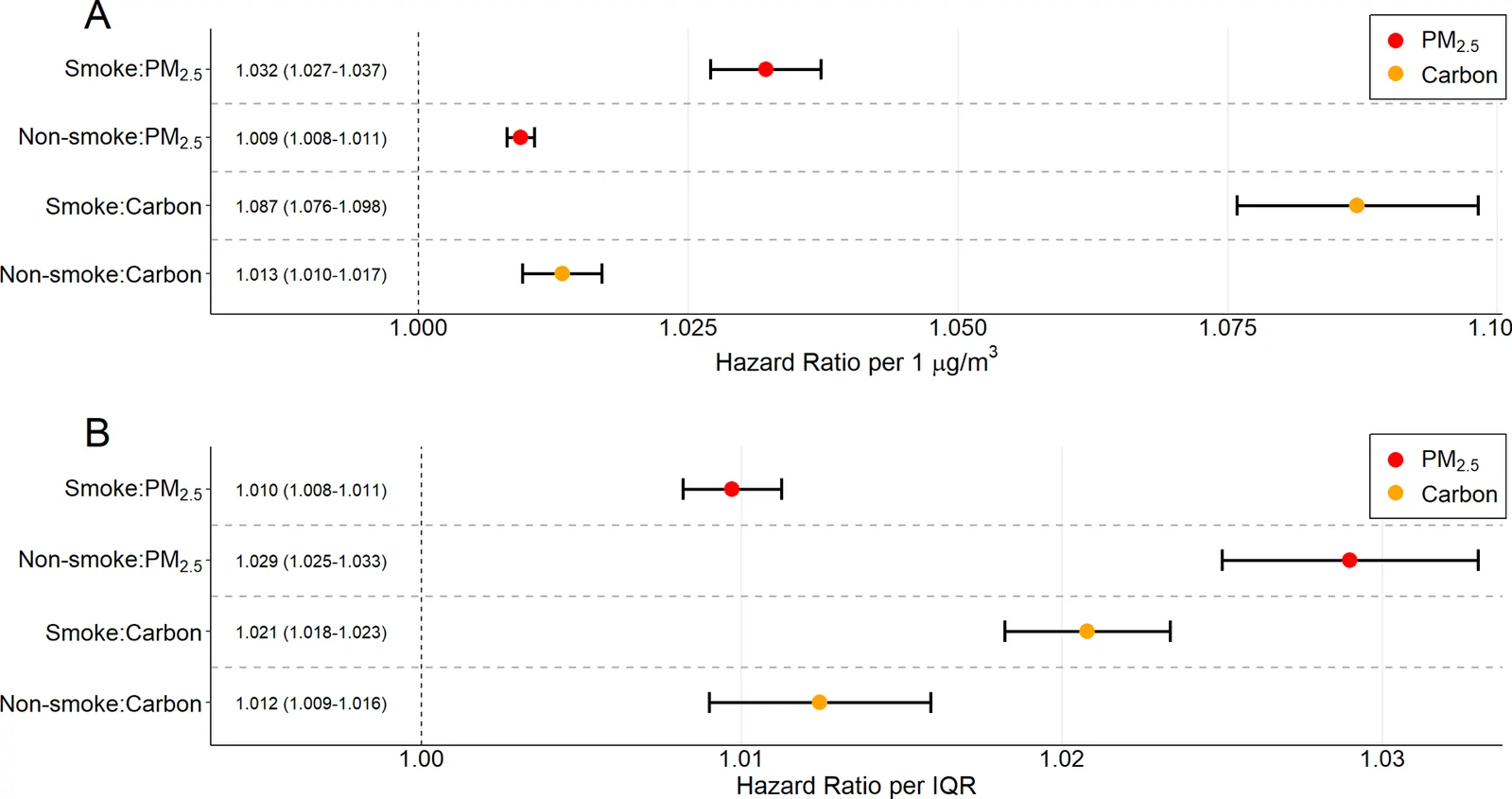
Hazard ratios (HRs) and 95% confidence intervals (CIs) of 3 year moving average smoke and non-smoke PM_2.5_ mass and carbon on all-cause mortality. (A) Per 1 μg/m^3^ increase. (B) Per IQR increase.

Exposure–response curves for smoke PM_2.5_ and smoke carbon rose steeply at low concentrations (<0.5 μg/m^3^ for PM_2.5_ and <0.15 μg/m^3^ for carbon) and then increased more gradually across intermediate ranges (0.50–1.25 μg/m^3^ for PM_2.5_ and 0.15–0.65 μg/m^3^ for carbon, Figure 3). At higher concentrations, HRs slightly declined and CIs widened, reflecting sparse data. Therefore, the upper-tail pattern did not provide clear evidence of saturation. The exposure–response curves for non-smoke PM_2.5_ and non-smoke carbon also showed nonlinear patterns (Figure S4).

**Figure 3:**
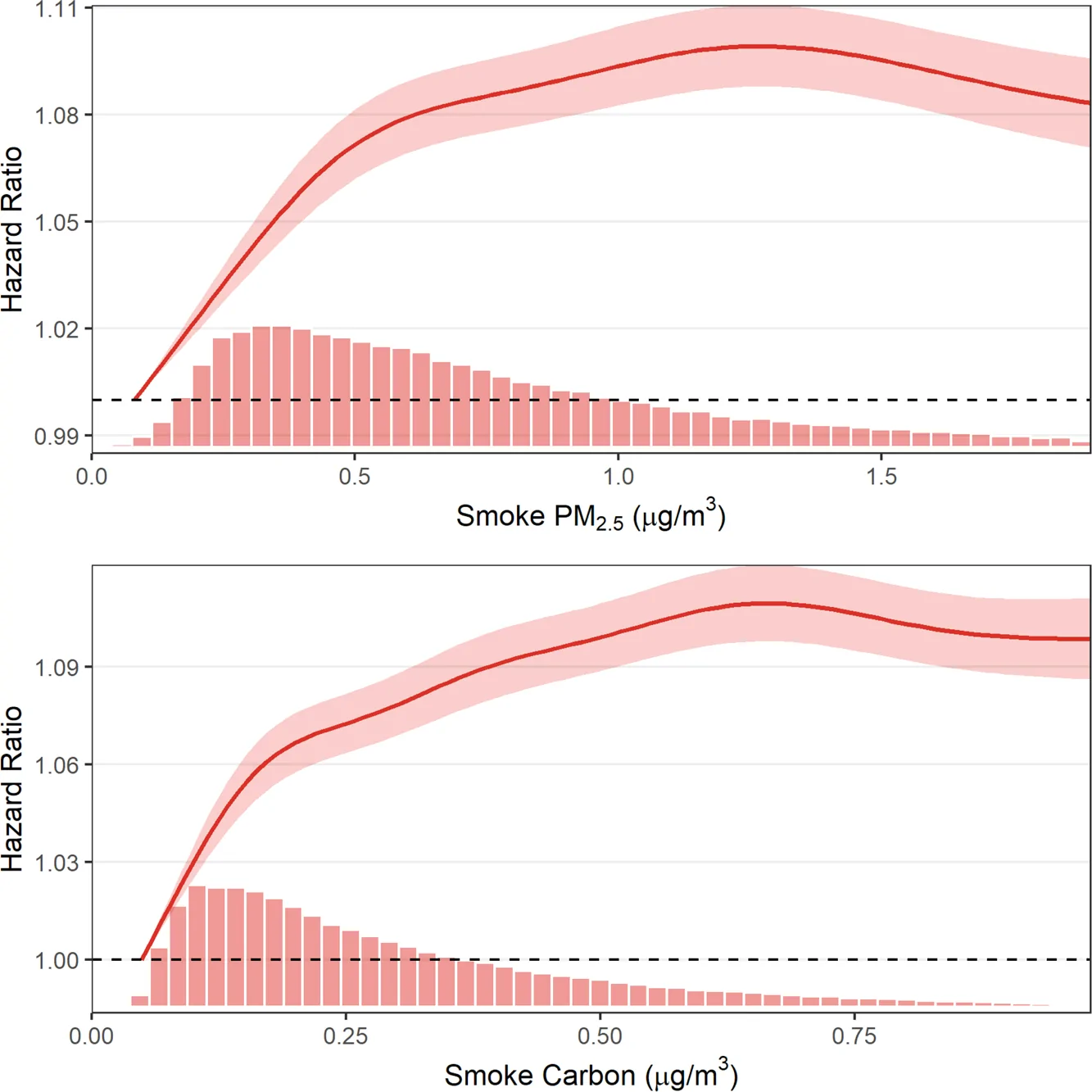
Exposure–response functions for 3 year moving-average concentrations of smoke PM_2.5_ mass and smoke carbon in relation to all-cause mortality. The concentrations span the 1st to the 99th percentiles for each pollutant.

### Subgroup and Sensitivity Analyses

In subgroup analyses, stronger associations were observed among Medicaid-eligible beneficiaries and Black and other racial groups, whereas associations did not differ significantly by sex. Geographic heterogeneity was evident, with weak or null associations in the regimes characterized by relatively high smoke contributions and more frequent heavy-smoke conditions (regimes 1–2) and larger effect estimates in regimes with lower smoke contributions and fewer heavy-smoke days that also differed in carbonaceous composition and meteorological characteristics (regimes 3–5). By the U.S. region, associations were strongest in the Northeast, followed by the Southeast, while estimates were weaker in the Midwest and West and null in the Southwest (Table 2). Sensitivity analyses showed attenuated associations for the 1 year and 2 year exposure windows and stronger associations for the 5 year exposure window. Associations were also smaller among nonmovers and among nonmovers residing in ZIP codes with low within-ZIP smoke variability. Estimates were slightly larger after excluding temperature and relative humidity and were attenuated when regimes 1 and 2 were combined. Smoke OC and EC showed larger per-unit-mass associations than smoke carbon, whereas the smoke and nonsmoke noncarbonaceous fractions showed weak positive associations. When smoke OC and EC were included simultaneously, either alone or with smoke noncarbonaceous PM_2.5_, both OC and EC remained positively associated with mortality. Associations were also larger when unweighted ZIP code exposures were used and were attenuated after additional adjustment for tobacco use disorder, alcohol use disorder, and drug use disorder (Table S8).

**Table 2:** Subgroup Analyses Hazard Ratios of 3 Year Moving Average Smoke PM_2.5_ Mass and Smoke Carbon on All-Cause Mortality per 1 μg/m^3^ Increase

modifier	group	hazard ratio per 1 μg/m^3^ (95% CI)^a^	
		smoke PM_2.5_	smoke carbon
sex	male	1.033 (1.028–1.039)	1.084 (1.072–1.097)
	female	1.032 (1.026–1.037)	1.090 (1.079–1.101)
	*P* value^a^	0.35	0.45
race	White	1.031 (1.026–1.037)	1.078 (1.056–1.101)
	Black	1.042 (1.029–1.055)	1.085 (1.073–1.097)
	Other	1.068 (1.055–1.080)	1.173 (1.143–1.204)
	*P* value	<0.001	<0.001
Medicaid eligibility	no	1.021 (1.016–1.026)	1.047 (1.037–1.058)
	yes	1.065 (1.056–1.075)	1.214 (1.192–1.237)
	*P* value	<0.001	<0.001
smoke regimes	1	0.995 (0.978–1.011)	1.001 (0.967–1.034)
	2	1.005 (0.988–1.023)	1.015 (0.975–1.056)
	3	1.045 (1.038–1.053)	1.136 (1.117–1.155)
	4	1.051 (1.041–1.060)	1.059 (1.042–1.075)
	5	1.057 (1.041–1.072)	1.084 (1.053–1.115)
	*P* value	<0.001	<0.001
U.S. regions	Midwest	0.990 (0.975–1.005)	1.046 (1.018–1.074)
	Northeast	1.267 (1.241–1.293)	1.489 (1.433–1.548)
	Southeast	1.034 (1.026–1.044)	1.061 (1.043–1.079)
	Southwest	1.010 (0.993–1.027)	1.011 (0.983–1.040)
	West	1.008 (1.001–1.016)	1.036 (1.018–1.054)
	*P* value	<0.001	<0.001

a The *P* value for interaction based on the Wald test comparing subgroup coefficients was reported as a complementary measure to summarize heterogeneity across subgroups.

### Attributable Mortality Burden

The mean annual mortality burden attributable to smoke PM_2.5_ among adults ≥65 years was 22,420 deaths, compared with 93,776 for non-smoke PM_2.5_ (Table S9). Smoke-attributable deaths increased over time, peaking at 40,554 in 2019, when exposures reflected a 3 year average that includes the 2017–2018 megafires. By contrast, non-smoke deaths declined from 122,705 in 2005 to 76,509 in 2019. On average, smoke carbon accounted for 93.1% of smoke PM_2.5_ deaths, with 6.9% attributed to the non-carbonaceous fraction. By state, mean annual smoke PM_2.5_ mortality rates were highest in Montana (226.9 deaths per 100,000 people, Figure 4), Idaho (166.3), Utah (124.8), Georgia (109.9), and Alabama (107.9), and mean annual attributable death counts were highest in California (2567 deaths, Table S10), Florida (1564), Texas (1476), Georgia (1277), and North Carolina (1158).

**Figure 4:**
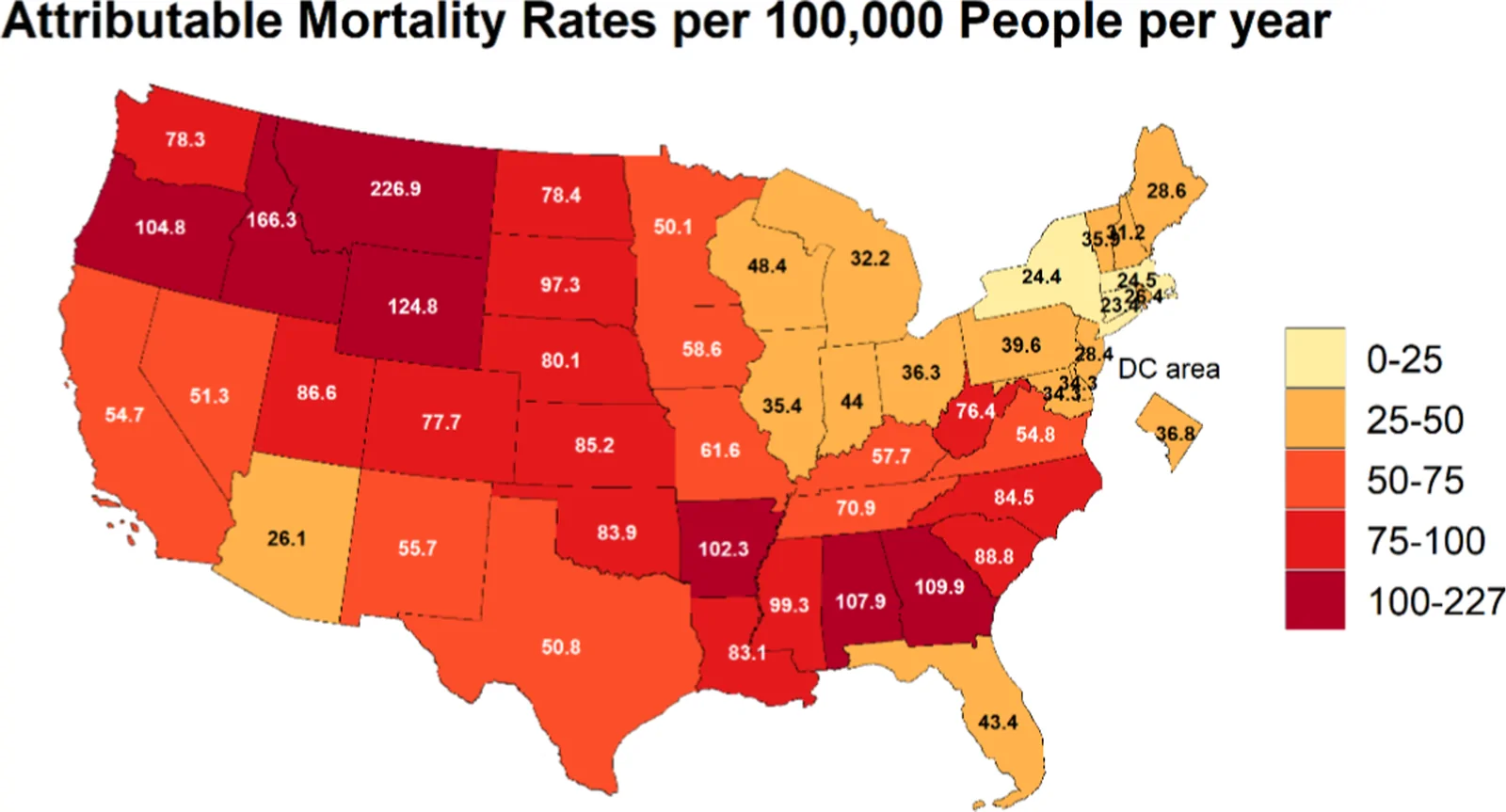
State-level annual mortality rates attributable to wildfire smoke PM_2.5_ mass, per 100,000 population.

## Discussion

In this study, we found that long-term exposure to smoke PM_2.5_ was associated with an increased risk of all-cause mortality, consistent with the limited smoke-specific studies on chronic mortality.^9,10,28^ In addition, our source-resolved analysis found stronger per-unit mass associations for smoke PM_2.5_ and its carbonaceous fraction than for their non-smoke counterparts. These comparisons should be interpreted cautiously because the exposure distributions differed substantially and a 1 μg/m^3^ increase represented a relatively large and less typical contrast for smoke. The exposure–response curves, IQR, 90IPR, and 0.1 μg/m^3^ estimates therefore provide complementary measures of associations across exposure contrasts more representative of the observed distributions. These findings are consistent with previous research comparing the effects of smoke and non-smoke PM_2.5_ on different health outcomes.^7,8,29,30^ Our study extended this evidence by providing speciation-resolved evidence that the carbonaceous fraction of smoke PM_2.5_ may explain the elevated mortality risk associated with wildfire smoke PM_2.5_.

A plausible explanation lies in the distinct composition of smoke PM_2.5_ with a higher carbonaceous fraction than non-smoke PM_2.5_ (in our data: 70.1% in smoke vs 32.9% in non-smoke) and greater toxicity of the smoke carbonaceous fraction. Carbonaceous matter has been demonstrated to be an important component influencing PM_2.5_ toxicity through multiple biological pathways and is a key contributor to mortality.^11,12,19,31^ We also found larger per-unit associations for smoke carbon than for its non-smoke counterpart, with consistent results for OC and EC in the sensitivity analyses. Mechanistically, biomass-burning OC is enriched in redox-active organics that generate reactive oxygen species and induce oxidative stress.^32–34^ Moreover, atmospheric aging (i.e., photochemical and heterogeneous processing) further increases oxygenated organic content and enhances the oxidative potential of smoke during long-range transport.^15,33^ Smoke EC provides a large sorptive surface area and organic coatings that efficiently adsorb semivolatile toxicants (e.g., PAHs), facilitating delivery to the respiratory tract.^34–36^ Consistent with this chemistry, biomass-burning particles exhibit elevated oxidative potential in acellular assays, and source-apportionment studies frequently attribute a substantial share of PM_2.5_ oxidative activity to biomass-burning contributions.^15,37^ In contrast, much of the oxidative potential of non-smoke mixtures arises from non-carbonaceous components such as traffic-related metals and acidity.^17,38^ Among non-smoke carbonaceous components, OC from traffic contains substantial petrogenic material from lubricating oil and fuel, including hydrocarbons, hopanes, and steranes.^39^ EC from biomass burning and fossil-fuel combustion also differ in their morphology, mixing state, surface coatings, and associated chemical species.^40^ These non-smoke carbonaceous particles are also associated with adverse health effects, although differences in physicochemical characteristics may contribute to the smaller per-unit mortality association observed for non-smoke carbon in our study.^41^ In addition, differences in particle-size distribution across smoke and non-smoke PM_2.5_ may contribute to different biological responses. Smaller particles can deposit efficiently in the distal respiratory tract and may induce systemic biological responses following inhalation.^42^ Biomass-combustion aerosol commonly contains substantial submicrometer particle fractions, although particle size varies according to fuel type, combustion conditions, and atmospheric aging.^43,44^ Smoke particles can also grow during transport through coagulation and condensation. Other combustion sources, particularly traffic and industrial emissions, also produce substantial submicrometer particles, including abundant ultrafine particles from fresh combustion.^45^ Together, differences in carbonaceous composition, emission sources, organic functionality, co-emitted toxicants, atmospheric aging, and particle size provide a biologically plausible rationale for the stronger per-unit mortality association observed for smoke carbon and carbon-rich smoke PM_2.5_.

Our non-smoke estimates (PM_2.5_ HR per 10 μg/m^3^: 1.098 [95% CI: 1.083–1.116], corresponding to the unrounded HR of 1.009 per 1 μg/m^3^ reported in Table S7) were slightly higher than prior U.S. national cohort studies of ambient PM_2.5_ and components. For example, both Di et al. (1.073 [1.071–1.075]) and Shi et al. (1.065 [1.057–1.073]) reported a lower all-cause mortality per 10 μg/m^3^ increase in annual PM_2.5_.^27,46^ Hao et al. observed associations for PM_2.5_ organic matter (HR per 1 μg/m^3^: 1.017–1.023) and black carbon (1.059–1.169).^11^ Prior studies relied on area-averaged exposures at the ZIP code level and shorter (1 year) exposure windows, whereas our models used population-weighted exposures and 3 year averages, which assign greater weight on higher exposures in densely populated areas and reflect cumulative health effects.

To further assess whether the mortality associations differed between carbonaceous and non-carbonaceous components, we examined the non-carbonaceous fraction of PM_2.5_ in sensitivity analyses. Results suggested weaker associations for the non-carbonaceous smoke PM_2.5_ than for smoke carbon, indicating that the stronger associations observed for smoke PM_2.5_ are more reflected by the carbonaceous fraction. Ambient PM_2.5_ without smoke influence is often dominated by sulfate and nitrate in the U.S.^18,47^ By comparison, wildfire smoke’s non-carbonaceous fraction is often enriched in ash and crustal/mineral material with tracers such as potassium and chloride.^48,49^ Epidemiological evidence more consistently links sulfate and nitrate to higher mortality risk, whereas associations for crustal or mineral components are generally weaker or heterogeneous.^11,12,50^ Sulfate and nitrate can indirectly amplify toxicity by lowering particle pH and increasing transition-metal solubility, thereby reinforcing metal-driven redox chemistry characteristic of non-smoke PM_2.5_.^51^

Subgroup analyses indicated higher risks associated with smoke PM_2.5_ among Black and other racial groups and Medicaid-eligible (lower-SES) beneficiaries. People of color and low-SES communities tend to experience poorer baseline health and less access to both medical resources and protective measures that reduce indoor smoke penetration.^52^ We also observed pronounced heterogeneity across smoke regimes, with larger mortality associations in regimes characterized by lower to moderate smoke contributions and greater influence of transported or aged smoke, whereas associations were null in regimes dominated by frequent, high-intensity smoke. This pattern suggests that chronic health risks from wildfire smoke depend not only on exposure magnitude but also on smoke carbon composition, exposure frequency, and atmospheric transport conditions. Consistent with these regime patterns, associations were strongest in the Northeast and Southeast, weaker in the West, and null in the Midwest and Southwest, partly reflecting differences in the distribution of smoke regimes across the regions. In addition, differences in adaptive capacity, including risk communication, access to filtration and clean-air spaces, and behavioral responses, may further contribute to these gradients.^53^ Although these regional patterns, particularly the weaker association in the west, are broadly consistent with prior studies, they should be interpreted with caution because substantial heterogeneity in smoke characteristics exists within regions.^7,8,54,55^ For example, in California, we identified smoke regimes representing both near-source wildfire smoke and predominantly transported smoke that exhibited different mortality associations despite occurring within the same state. These findings suggest that wildfire smoke mitigation and health protection strategies may need to be applied according to regional smoke characteristics, with greater emphasis on managing transported and aged smoke in downwind populations.

The attributable-burden analysis indicated that wildfire smoke represents an increasing share of the long-term PM_2.5_-related mortality burden among older U.S. adults, indicating that wildland fire smoke is emerging as a major public-health burden. Although smoke contributes a smaller share of the total PM_2.5_ mass, its higher toxicity and widespread plumes yield a substantial burden. By contrast, nonsmoke PM_2.5_ pollution has declined with national air quality improvement from on-road controls, power generation, and other sectors.^56^ It is essential to integrate wildfire smoke into air quality and climate adaptation planning and to prioritize mitigation in regions where smoke drives the largest mortality.

Our study has several strengths. First, to our knowledge, it is the first large, nationwide cohort-based analysis of mortality associated with long-term exposure to smoke-specific PM_2.5_ mass and its carbonaceous components. Second, the large cohort (∼77 million) provided substantial statistical power and enabled systematic evaluation of effect modification by sex, race, SES, smoke regimes, and regions. Third, we used high spatiotemporal resolution exposure estimates that explicitly distinguished smoke and non-smoke sources and provided OC/EC speciation. This approach greatly improved exposure characterization compared to using crude proxy metrics such as “smoke days” or plume masks. Fourth, we developed and applied data-driven smoke regime classification that characterized ZIP codes according to long-term heterogeneity in smoke contribution, composition, frequency, and atmospheric transport conditions. This approach complemented the geographic region classification by grouping geographically separated ZIP codes with similar smoke environments and distinguishing different smoke profiles within broad geographic regions. It therefore allowed evaluation of heterogeneity that may be obscured when wildfire smoke is treated as a spatially uniform exposure. Finally, our effect estimates for smoke PM_2.5_ can inform health-cost accounting for wildfires and the cost-effectiveness of fuels management and smoke protection measures. Future studies could extend these analyses to cause-specific mortality such as cardiovascular and respiratory mortality, providing a more complete assessment of the health burden associated with long-term exposure to smoke-specific PM_2.5_ and its carbonaceous components.

Several limitations must be acknowledged. First, exposures were assigned at the ZIP code level rather than at residential addresses, which likely introduced exposure measurement error. Although the positive pattern persisted across our sensitivity analyses, these findings demonstrate that the magnitude of the associations was sensitive to exposure assignment assumptions. Second, we did not directly resolve specific inorganic species (e.g., sulfate, nitrate, dust), so results about non-carbonaceous PM_2.5_ must be interpreted with caution. Finer-speciated exposure products will be needed to test this interpretation more definitively. Third, we did not adjust for wildfire-related gaseous pollutants, including O_3_, NO_2_, and VOCs, because long-term, high-resolution, and source-resolved exposure estimates with national coverage were unavailable. These pollutants may co-occur with wildfire smoke and contribute independently or jointly to adverse health effects. Consequently, the estimated associations for smoke PM_2.5_ may partly reflect the broader wildfire pollution mixture. Fourth, individual behavioral and clinical risk factors were not available and were addressed with area-level proxies. In a sensitivity analysis, additional adjustment for claims-based tobacco-use disorder, alcohol-use disorder, and drug-use disorder attenuated the associations. These diagnoses likely capture more severe or clinically recognized cases and may not represent the full range of the behavioral patterns. Although a previous Medicare-based study reported that the absence of adjustment for individual-level risk factors does not materially bias PM_2.5_-mortality associations, residual confounding by incompletely measured behavioral and clinical factors cannot be excluded.^27^ Fifth, because the cohort consisted of Medicare beneficiaries aged 65 years or older, the findings are directly generalizable to older U.S. adults but may not quantitatively represent associations among younger populations. Younger adults may differ in baseline health status, occupational exposure, and physiological susceptibility to smoke, which may result in different exposure–response relationships. Sixth, the smoke regimes were fixed over time and derived from modeled exposures, making them sensitive to exposure uncertainties and clustering methodologies. In addition, some wildfire and smoke characteristics, such as the ignition type, fuel composition, smoke age, and transport distance, were not directly included. Future studies should evaluate more comprehensive regime classifications.

## Supplementary Material



## Data Availability

Medicare data are not publicly available and are subject to CMS data-use agreements. Access may be obtained through CMS following its standard application procedures.

## Notes

The Supporting Information includes the construction of the smoke-regime indicator (Section S1), details of the sensitivity analyses (Section S2), attributable mortality burden estimation (Section S3), and supplementary Tables S1–S10 and Figures S1–S4 (PDF)
