# Supplementary material for: Carbonaceous Components Underlying Wildfire Smoke PM2.5-Associated Mortality in Older U.S. Adults
Source: Environ Sci Technol. 2026 Sep 30;60(40):28397–407. doi: 10.1021/acs.est.6c07219 (PMC13674056; doi:10.1021/acs.est.6c07219)
Supplement: Supplementary file 1 [file es6c07219_si_001.pdf]

Supplementary Appendix

Carbonaceous components underlying wildfire smoke $PM_{2.5}$-associated mortality in older U.S. adults

Zhihao Jin[1], Ke Xu[1], Hua Hao[1], Meng Qi[1], Gonzalo A. Ferrada[2], Joshua S. Fu[2,3], Noah Scovronick[1], Howard H. Chang[4], Alvaro Alonso[5], Kyle Steenland[1], Yang Liu[1*]

[1]Gangarosa Department of Environmental Health, Rollins School of Public Health, Emory University, Atlanta, GA 30322, USA

[2]Department of Civil and Environmental Engineering, University of Tennessee, Knoxville, TN 37996, USA

[3]Integrated Computational Earth Sciences Group, Computational Sciences and Engineering Division, Oak Ridge National Laboratory, Oak Ridge, TN 37381, USA

[4]Department of Biostatistics and Bioinformatics, Rollins School of Public Health, Emory University, Atlanta, GA 30322, USA

[5]Department of Epidemiology, Rollins School of Public Health, Emory University, Atlanta, GA 30322, USA

*Corresponding author: Yang Liu, Email: yang.liu@emory.edu, address: 1518 Clifton Rd NE, Atlanta, GA 30322

Supplementary Information

Section S1. Smoke regime dummy variable
Section S2. Sensitivity analyses
Section S3. Attributable mortality burden

Supplementary Tables

Table S1. Exposure modeling performance based on 10-fold random cross-validation.
Table S2. Pearson correlation between concentrations of smoke and non-smoke $PM_{2.5}$, OC, and EC at annual 1-km level.
Table S3. Descriptive statistics for the population and geographic area of ZIP codes included in the study (2002-2019).
Table S4. Distributions of 3-year moving average exposure levels (unit: $\mu g/m^3$).
Table S5. Validation of candidate Ward hierarchical clustering solutions for ZIP-code-level smoke regimes.
Table S6. Mean values of variables used in defining smoke regimes.
Table S7. Hazard ratios (HRs) and 95% confidence intervals (CIs) of smoke and non-smoke $PM_{2.5}$ mass and carbon on all-cause mortality with per 1 $\mu g/m^3$, per 0.1 $\mu g/m^3$, per IQR, and per 90IPR increase.
Table S8. Sensitivity analyses results.
Table S9. Annual attributable deaths for non-smoke and smoke $PM_{2.5}$ among U.S. older adults (2005-2019), with allocation to carbonaceous and non-carbonaceous fraction.
Table S10. State-level annual attributable mortality rates (per 100,000) and attributable deaths for non-smoke and smoke $PM_{2.5}$ among U.S. older adults (2005-2019), with allocation to carbonaceous and non-carbonaceous fraction.

Supplementary Figures

Figure S1. Five smoke regimes by ZIP codes. ZIP codes with the same color belong to the same smoke regime.
Figure S2. Five U.S. geographical regions used in this study (Northeast, Southeast, Midwest, West, Southwest).
Figure S3. Mean non-smoke (left) and smoke (right) OC-to-EC ratio across the CONUS, 2002-2019. Mapped with 1-km spatial resolution.
Figure S4. Exposure-response functions for 3-year moving-average concentrations of non-smoke $PM_{2.5}$ mass and non-smoke carbon in relation to all-cause mortality. The concentrations span the 1st to the 99th percentiles for each pollutant.

Supplementary Information

S1. Smoke regime dummy variable

To account for broad-scale spatial heterogeneity in wildfire smoke contribution, composition, frequency, and atmospheric transport conditions, we constructed a ZIP code-level categorical variable characterizing long-term smoke regimes. Although fire type, fuel conditions, plume transport distance, and regional climate were not measured directly, the variables used to define the smoke regimes provided indirect information on these smoke characteristics. For each ZIP code, we calculated long-term averages (2002-2019) of smoke $PM_{2.5}$ mass and its carbonaceous component concentrations. Smoke contribution was expressed as the proportion of smoke $PM_{2.5}$ relative to total $PM_{2.5}$, and smoke composition was expressed as the proportional contribution of carbonaceous components within total smoke $PM_{2.5}$; we also included the ratio of smoke OC to smoke EC. These metrics characterize differences in the relative influence and chemical composition of combustion emissions independently of absolute concentrations. Frequency of heavy smoke day was defined as the fractions of annual smoke days exceeding prespecified smoke $PM_{2.5}$ thresholds (>1 and >2.5 $\mu g/m^3$) relative to annual smoke days (>0 $\mu g/m^3$). Atmospheric transport and dispersion conditions were characterized using long-term mean wind speed derived from the zonal and meridional components, planetary boundary-layer height, and precipitation, which are derived from the ECMWF reanalysis v5 (ERA-5) and North American Regional Reanalysis (NARR) datasets. These variables provided information on long-term differences in smoke occurrence, dispersion, and atmospheric transport conditions. All clustering variables were standardized to have mean zero and unit variance. ZIP codes were then grouped using hierarchical clustering with Ward's minimum-variance method and Euclidean distance. Although the specific smoke-regime framework developed in this study has not been adopted in wildfire epidemiology, clustering methods have been used in air pollution epidemiology to identify locations or time periods with similar multipollutant profiles and to evaluate whether health associations differ across pollution mixtures.[1]

We evaluated candidate solutions containing two to eight clusters using total within-cluster sum of squares, average silhouette width, the Calinski-Harabasz index, and scientific interpretability. Solutions with two or three clusters had higher silhouette widths and Calinski-Harabasz indices but were highly imbalanced, with most ZIP codes assigned to the largest cluster, and combined several distinguishable smoke exposure patterns. The five-cluster solution explained 38.8% of the total variation and identified five interpretable profiles characterized by differences in smoke contribution, carbonaceous composition, smoke frequency, and meteorological conditions. Although solutions with more clusters modestly improved silhouette width and explained variation, they primarily subdivided an existing regime without identifying an additional scientifically distinct exposure profile. We therefore selected five clusters as a balance between parsimony, statistical performance, cluster balance, and scientific interpretability. Each ZIP code was assigned to a single smoke regime that remained fixed over the study period. Because smoke regimes were defined based on similarity in long-term contribution, composition, frequency, and atmosphere transport context rather than geographic proximity, regime assignments were not constrained to be spatially contiguous.

The five smoke regime categories exhibited distinct and interpretable differences in smoke contribution, composition, frequency, and atmospheric transport conditions (Figure S1; Table

S6). Regime 1 was characterized by the highest contribution of smoke $PM_{2.5}$ to total $PM_{2.5}$ and a large proportion of heavy smoke days, with a moderate fraction of carbonaceous matter, occurring under relatively dry conditions with high boundary-layer heights. This pattern may be consistent with persistent, intense near-source smoke influence and a complex mixture of combustion products. This interpretation is also consistent with the geography of wildfire-prone landscapes, which often encompass rural and sparsely populated areas.[2] Thus, Regime 1 includes a notable number of ZIP codes while representing only a small proportion of study participants. Regime 2 showed moderate smoke contribution and a lower frequency of heavy smoke days than Regime 1. Its carbon-rich smoke composition, higher wind speed, higher boundary-layer heights, low precipitation, and geographic proximity to Regime 1 are consistent with frequent exposure to short-range transported smoke. Regime 3 was least impacted by wildfire smoke, with the lowest smoke contribution and the lowest frequency of heavy smoke days, consistent with predominantly background $PM_{2.5}$ produced by human activities. When smoke was present, higher precipitation and lower boundary-layer heights correspond to more diluted, long-range transported smoke from the western U.S. and Canada. Regime 4 was characterized by frequent smoke days but low smoke contribution to $PM_{2.5}$, elevated elemental carbon fractions, and greater precipitation, suggesting frequent low-intensity smoke exposure likely from domestic prescribed fires. Regime 5 represented a transitional regime with intermediate smoke contribution and composition similar to Regime 4, but showed stronger winds and distinct wind patterns, consistent with recurrent exposure to aged or transported smoke plumes influenced by regional transport and domestic prescribed fire smoke. Overall, Regimes 1 and 2 represented patterns consistent with relatively fresh, near-source, or short-range transported smoke, whereas Regimes 3 to 5 reflected more diluted, aged, or regionally transported smoke with fewer heavy-smoke days, including recurrent influences from domestic prescribed burning.

S2. Sensitivity analyses

We conducted a series of sensitivity analyses to evaluate the robustness of the primary findings to alternative exposure windows, potential exposure misclassification, meteorological adjustment, smoke-regime classification, component specification, ZIP-code exposure aggregation, and residual confounding by available individual-level behavioral indicators. Unless otherwise specified, all sensitivity analyses retained the cohort definition, underlying time scale, covariate adjustment, and model specification used in the primary analyses.

S2.1 Alternative exposure averaging windows

The primary analyses used annually updated 3-year moving-average concentrations of smoke $PM_{2.5}$ and smoke carbon. To evaluate the cumulative effects of smoke and whether the findings were sensitive to the selected exposure window, we repeated the primary models using 1-year, 2-year, and 5-year moving-average concentrations for both exposures.

S2.2 Residential mobility and within-ZIP exposure variability

Because exposures were assigned according to annually updated residential ZIP codes rather than precise residential addresses, we performed two analyses to evaluate potential exposure misclassification. First, we restricted the cohort to non-movers, defined as beneficiaries whose residential ZIP code remained unchanged throughout follow-up. This restriction reduced exposure misclassification arising from residential mobility and uncertainty regarding the timing of address changes.

Second, we further restricted the non-mover population to ZIP codes with relatively low within-ZIP spatial variability in smoke exposure. We used annual 1-km estimates of smoke $PM_{2.5}$ and smoke carbon from 2002 to 2019. For each ZIP code and calendar year, we calculated the population-weighted standard deviation of the 1-km exposure estimates using annual LandScan population counts as weights. We then averaged the annual standard deviations across 2002 to 2019 to obtain a long-term measure of within-ZIP spatial variability for each exposure.

Because absolute within-ZIP variability was positively associated with the long-term mean exposure concentration, ZIP codes were grouped into deciles of their long-term mean exposure. Within each decile, ZIP codes with long-term within-ZIP standard deviations below the decile-specific median were classified as having low spatial variability. This classification was conducted separately for smoke $PM_{2.5}$ and smoke carbon. We then repeated the corresponding primary Cox model among beneficiaries who did not change residential ZIP codes during follow-up and who resided in ZIP codes classified as having low variability for the exposure under evaluation. All other aspects of the model specification and covariate adjustment were retained.

S2.3 Population weighting of ZIP-code exposures

The primary ZIP-code exposure estimates were calculated as population-weighted means of the underlying 1-km grid-cell concentrations using LandScan population data. To evaluate sensitivity to this weighting procedure and to facilitate comparison with studies using spatially unweighted ZIP-code exposures, we repeated the primary analyses using the arithmetic mean of all 1-km exposure grid cells within each ZIP code without population weighting.

S2.4 Meteorological adjustment

The primary models adjusted for annually updated 3-year moving-average temperature and relative humidity to account for long-term spatial and temporal variation in meteorological conditions and to align the meteorological covariates with the primary exposure window. To evaluate whether the findings depended on this adjustment, we repeated the primary models after excluding both temperature and relative humidity while retaining all other covariates.

S2.5 Alternative smoke-regime classification

The primary analysis used five empirical smoke regimes that characterized ZIP codes according to long-term smoke contribution, carbonaceous composition, smoke-event frequency, and selected meteorological conditions. Regime 1 represented a relatively small proportion of the cohort but was environmentally distinct, with the highest contribution of smoke $PM_{2.5}$ to total $PM_{2.5}$ and the greatest frequency of very heavy smoke days. To evaluate whether the findings were sensitive to retaining Regime 1 as a separate category, we combined Regimes 1 and 2 and repeated the primary models using the resulting four-regime classification. Regimes 1 and 2 were selected for combination because both were characterized by relatively high smoke contributions and frequent heavy-smoke conditions, although they differed in other compositional and meteorological characteristics.

S2.6 Component-specific exposure models

To examine associations with individual carbonaceous and non-carbonaceous components, we first fitted separate models for smoke OC, smoke EC, and smoke non-carbonaceous $PM_{2.5}$. Smoke non-carbonaceous $PM_{2.5}$ was calculated as smoke $PM_{2.5}$ mass minus smoke carbon, where smoke carbon was defined as the sum of smoke OC and smoke EC. Corresponding separate models were also fitted for the non-smoke components. We subsequently fitted two

mutually adjusted smoke-component models. The first included smoke OC and smoke EC simultaneously. The second included smoke OC, smoke EC, and smoke non-carbonaceous $PM_{2.5}$ simultaneously. These models were intended to estimate the association of each modeled component conditional on the other included components. Because the components were correlated and jointly contributed to the total smoke $PM_{2.5}$ mixture, the resulting coefficients were interpreted as conditional associations rather than as fully independent causal effects. All component analyses used annually updated 3-year moving-average concentrations and retained the covariate adjustment used in the primary models.

S2.7 Claims-based behavioral indicators

Detailed individual-level information on smoking history, alcohol consumption, drug use, and other behavioral factors was unavailable in the Medicare data. To evaluate potential residual confounding using the available individual-level claims information, we repeated the primary models with additional adjustment for indicators of tobacco use disorder, alcohol use disorder, and drug use disorder obtained from the CCW Other Chronic or Potentially Disabling Conditions segment. These indicators were restricted to sensitivity analyses because their identification windows differed from those used for the standard annual CCW covariates. In addition, claims-based diagnoses are likely to capture more severe or clinically recognized cases rather than the full distribution of tobacco, alcohol, and drug use within the Medicare population.

S3. Attributable mortality burden

We quantified county–year mortality attributable to smoke and non-smoke $PM_{2.5}$ using a coherent counterfactual and parametric Monte Carlo simulation (10,000 iterations). For each county and year, we (i) computed the population attributable fraction (PAF), defined as the proportion of deaths that would be avoided if smoke and non-smoke $PM_{2.5}$ were reduced from their observed levels to a low counterfactual level; (ii) combined the smoke and non-smoke PAFs into a total $PM_{2.5}$ PAF; and (iii) used a Shapley (average attributable fraction) decomposition to allocate the total $PM_{2.5}$ burden first between smoke and non-smoke $PM_{2.5,}$ and (iv) allocated within the smoke $PM_{2.5}$, among carbonaceous and the non-carbonaceous fraction.

When multiple, correlated exposures contribute to the same deaths (e.g., smoke and non-smoke $PM_{2.5}$, or carbon, and non-carbonaceous smoke), simple single-exposure PAFs do not sum to the combined burden and can even exceed 100%. To avoid double-counting, we used a Shapley (average attributable fraction) decomposition, which is equivalent to the "average attributable fraction" proposed by Gefeller and Eide.[3] The Shapley idea is to imagine all possible orders of eliminating the exposures (e.g., eliminate smoke first, then non-smoke versus eliminate non-smoke first, then smoke).[4] For each order, we compute how many deaths are prevented when we remove a given exposure at the point it is switched off, after accounting for any exposures already removed. The Shapley share for an exposure is the average of these marginal contributions across all orders. This method has three properties: it treats exposures symmetrically (no arbitrary hierarchy), the component shares sum exactly to the total PAF (no double-counting), and if an exposure has no effect, it receives zero share. In practical terms, the Shapley share for smoke $PM_{2.5}$ can be interpreted as the expected fraction of deaths that would be prevented by eliminating smoke $PM_{2.5}$ if the order in which smoke and non-smoke $PM_{2.5}$ are eliminated were chosen at random. Within the smoke $PM_{2.5}$ share, we apply the same logic to decompose the smoke burden into contributions from smoke carbon, and non-carbonaceous smoke.

Let D_{ct} denote observed all-cause deaths in county c, year t. For smoke $PM_{2.5}$, C_{ct}^{SMK}, we used a counterfactual of no smoke contribution (0 μg/m³). For non-smoke $PM_{2.5}$, C_{ct}^{NS}, we defined a theoretical minimum-risk exposure level (TMREL) following the Global Burden of Disease (GBD) comparative risk assessment framework, in which TMREL is represented as a uniform distribution over low long-term concentrations.[5] To tailor this to the U.S. exposure distribution, we specified a TMREL T_s drawn from a uniform distribution on [2.4, $P_{1^{st}}$] μg/m³ for each iteration s:

$$T_s \sim Unif(2.4, P_{1^{st}})$$

Where 2.4 μg/m³ is the minimum $PM_{2.5}$ concentration used in the GBD assessment framework and $P_{1^{st}}$ is the first percentile of 3-year moving average non-smoke $PM_{2.5}$ in our study (3.624 μg/m³). We compared each county–year's non-smoke concentration to this counterfactual, using the excess above TMREL:

$$C_{ct}^{NS,+} = \max\{C_{ct}^{NS} - T_s, 0\}$$

For each iteration s, we obtained the coefficients from the Cox models: β_{smk} for smoke $PM_{2.5}$, β_{ns} for non-smoke $PM_{2.5}$, and β_A, β_B, β_C for smoke carbon, and non-carbonaceous matter. For county-year and iteration, we draw log-slopes from normal distributions implied by the 95% CI:

$$\beta_{smk}^{(s)} \sim N(\hat{\beta}_{smk}, SE_{smk}^2)$$

$$\beta_{non-smoke}^{(s)} \sim N(\hat{\beta}_{non-smoke}, SE_{non-smoke}^2)$$

$\beta_A^{(s)}$,$\beta_B^{(s)}$,$\beta_C^{(s)}$ were drawn similarly. The smoke PAF is：

$$PAF_{smk,ct}^{(s)} = 1 - \exp\{-\beta_{smk}^{(s)} C_{ct}^{SMK}\}$$

and the non-smoke PAF (relative to TMREL) is:

$$PAF_{ns,ct}^{(s)} = 1 - \exp\{-\beta_{ns}^{(s)} C_{ct}^{NS,+}\}$$

Because PAF_{smk} and PAF_{ns} are not additively separable, we first combined smoke and non-smoke on the relative-risk (multiplicative) scale so that the total fraction is:

$$PAF_{tot,ct}^{(s)} = 1 - (1 - PAF_{smk,ct}^{(s)})(1 - PAF_{ns,ct}^{(s)}).$$

We then apportioned the total between smoke and non-smoke using the Shapley attribution based on the PAF function:

$$f(smk,ns) = 1 - \exp\{-(smk + ns)\}$$

with doses $smk = \beta_{smk}^{(s)} C_{ct}^{SMK}$ and $ns = \beta_{ns}^{(s)} C_{ct}^{NS,+}$. For each pollutant, its Shapley share ϕ is the average marginal contribution $f(S \cup \{j\} - f(S))$ over all orders of removing $\{smk,ns\}$.The Shapley share of smoke is the average marginal contribution over the $2! = 2$ coalition orders:

$$\phi_{smk,ct}^{(s)} = \frac{1}{2}[f(smk,0) - f(0,0)] + \frac{1}{2}[f(smk,ns) - f(0,ns)] = \frac{1}{2}(1 - e^{-smk})(1 + e^{-ns})$$

And the non-smoke Shapley share is $\phi_{ns,ct}^{(s)} = PAF_{tot,ct}^{(s)} - \phi_{smk,ct}^{(s)}$. ϕ is the assigned share of the pollutant, not a standalone PAF for that pollutant. Deaths attributable to smoke and non-smoke $PM_{2.5}$ are:

$$AD_{smk,ct}^{(s)} = \phi_{smk,ct}^{(s)} D_{ct}$$
$$AD_{ns,ct}^{(s)} = \phi_{ns,ct}^{(s)} D_{ct}$$

Within the smoke $PM_{2.5}$ portion, we further decomposed the burden into contributions from carbon, and the non-carbonaceous fraction using the Shapley attribution. For any subset $S \subseteq \{A,B$, define the component value function:

$f_\theta(S) = 1 - \exp\left(-\sum_{j \in S} \theta_j\right)$where $\theta_A = \beta_A C_{ct}^A, \theta_B = \beta_B C_{ct}^B$. For each smoke component, its provisional Shapley share $\tilde{\phi}$ is the average marginal contribution over all $2! = 2$ orders of removing $\{A,B\}$:

$$\tilde{\phi}_{A,ct}^{(s)} = \frac{1}{2}\left[f(\{\theta_A,0,0\}) - f(0,0,0)\right] + \frac{1}{2}\left[f(\{\theta_A,\theta_B\}) - f(0,\theta_B)\right]$$
$$\tilde{\phi}_{A,ct}^{(s)} = \frac{1}{2}(1 - e^{-\theta_A})[1 + e^{-\theta_B}]$$

with analogous expressions for $\tilde{\phi}_B$. Let provision total PAF implied by the components HRs:

$$\widetilde{PAF}_{smk,ct}^{(s)} = 1 - e^{-(\theta_A + \theta_B)}$$

We then scaled the provision shares so the sum equals to the burden attributed to smoke $PM_{2.5}$. The final Shapley shares were scaled as:

$$\phi_{A/B}^{(s)} = \tilde{\phi}_{A/B}^{(s)} \frac{\phi_{smk,ct}^{(s)}}{\widetilde{PAF}_{smk,ct}^{(s)}}$$

By construction, $\phi_A^s + \phi_B^s = \phi_{smk,ct}^{(s)}$ (no overlap). The deaths attributable to each smoke component are $AD_{A,ct}^{(s)} = \phi_{A,ct}^{(s)} D_{ct}$, etc.

Uncertainty was propagated via Monte Carlo draws with 10,000 iterations. For each iteration we sampled log-slopes and drew T_s for TMREL as above. We computed county–year PAFs, Shapley shares, and attributable deaths, and then aggregated to states and nationally. We report the posterior mean and 95% uncertainty intervals (2.5th-97.5th percentiles across draws).

Supplementary Tables

Table S1. Exposure modeling performance based on 10-fold random cross-validation.

**$PM_{2.5}$ Speciation**	**Wildfire Scenario**	**Daily (SL)**		**Yearly (RASL)**	
		RMSE	R^2	RMSE	R^2
$PM_{2.5}$ mass	Non-smoke	2.145	0.893	1.065	0.939
	Smoke-impacted	2.651	0.868	2.170	0.901
OC	Non-smoke	0.560	0.774	0.118	0.980
	Smoke-impacted	1.202	0.666	0.128	0.979
EC	Non-smoke	0.171	0.801	0.039	0.980
	Smoke-impacted	0.241	0.712	0.036	0.985

Abbreviation: SL: super-learner; RASL: residual-adjusted super-learner; R^2: coefficient of determination.

Table S2. Pearson correlation between concentrations of smoke and non-smoke PM2.5, OC, and EC at annual 1-km level.

	Smoke $PM_{2.5}$	Smoke OC	Smoke EC	Non-smoke $PM_{2.5}$	Non-smoke OC	Non-smoke EC
Smoke $PM_{2.5}$	/	0.784	0.543	-0.262	-0.143	-0.214
Smoke OC	0.784	/	0.751	-0.301	-0.193	-0.291
Smoke EC	0.543	0.751	/	-0.064	0.110	-0.014
Non-smoke $PM_{2.5}$	-0.262	-0.301	-0.064	/	0.779	0.747
Non-smoke OC	-0.143	-0.193	0.110	0.779	/	0.912
Non-smoke EC	-0.214	-0.291	-0.014	0.747	0.912	/

Table S3. Descriptive statistics for the population and geographic area of ZIP codes included in the study (2002-2019).

**ZIP-code characteristic**	**Mean**	**SD**	**Min**	**25%**	**50%**	**75%**	**Max**
Landscan Population	10034	14867	0	805	3114	13852	533330
Medicare Benficaries	1115	1727	0	93	318	1385	41748
Land Area (km^2)	252	617	< 0.01	32	105	249	26541

Table S4. Distributions of 3-year moving average exposure levels (unit: $\mu g/m^3$).

**3-year Average Exposure[1]**	**1%**	**5%**	**25%**	**50%**	**75%**	**95%**	**99%**
Smoke $PM_{2.5}$	0.080	0.117	0.206	0.319	0.511	1.028	1.898
Smoke $PM_{2.5}$ Carbon	0.049	0.077	0.143	0.234	0.389	0.721	0.982
Smoke OC	0.039	0.064	0.118	0.196	0.325	0.598	0.822
Smoke EC	0.004	0.009	0.021	0.037	0.065	0.128	0.183
Non-smoke $PM_{2.5}$	5.085	6.119	7.824	9.197	10.861	13.595	15.246
Non-smoke $PM_{2.5}$ Carbon	1.354	1.853	2.472	2.910	3.408	4.473	6.469
Non-smoke OC	1.129	1.498	1.932	2.217	2.517	3.336	4.893
Non-smoke EC	0.216	0.325	0.515	0.684	0.890	1.287	1.646

[1]Exposure were population-weighted based on the LandScan global population database.

Table S5. Validation of candidate Ward hierarchical clustering solutions for ZIP-code-level smoke regimes.

Number of clusters	Within-cluster SS	Between-cluster variation explained, %	Average silhouette width	Calinski-Harabasz index
2	338410	15.7	0.288	7505
3	303840	24.4	0.261	6464
4	274293	31.7	0.114	6215
**5**	**253971**	**38.8**	**0.139**	**5838**
6	233960	41.8	0.141	5756
7	221756	44.8	0.114	5429
8	209973	47.7	0.119	5236

Note: WSS, within-cluster sum of squares. Lower WSS indicates greater within-cluster homogeneity, although WSS necessarily decreases as the number of clusters increases. Between-cluster variation explained was calculated as the between-cluster sum of squares divided by the total sum of squares. Average silhouette width ranges from -1 to 1, with larger values indicating greater within-cluster cohesion and between-cluster separation. Larger Calinski-Harabasz index values indicate greater between-cluster separation relative to within-cluster dispersion.

Table S6. Mean values of variables used in defining smoke regimes.

Variables	Smoke Regime				
	1	2	3	4	5
% Smoke $PM_{2.5}$[1]	29.37%	10.90%	6.38%	8.00%	8.11%
% Smoke Carbon[2]	45.92%	50.47%	43.26%	52.50%	51.61%
Smoke OC:EC	8.49	7.23	7.08	4.53	6.75
% Heavy smoke days (1-2.5 μg/m^3)[3]	24.68%	38.54%	34.03%	40.33%	37.86%
% Heavy smoke days (> 2.5 μg/m^3)[3]	56.29%	33.54%	20.33%	23.61%	20.11%
Wind speed: zonal (m/s)	0.57	0.54	0.62	0.21	-0.27
Wind speed: meridional (m/s)	0.28	0.42	0.20	0.17	1.05
Annual precipitation (mm)	1028	492	1428	1600	1219
Planetary boundary-layer height (m)	1495.57	1687.12	1117.67	1189.59	1245.48

[1]relative to total $PM_{2.5}$ concentration
[2]relative to smoke $PM_{2.5}$ concentration
[3]relative to total smoke days

Table S7. Hazard ratios (HRs) and 95% confidence intervals (CIs) of smoke and non-smoke $PM_{2.5}$ mass and carbon on all-cause mortality with per 1 μg/m^3, per 0.1 μg/m^3, per IQR, and per 90IPR increase.

**Pollutants**	**Hazard Ratio (95% CI)**			
	Per 1 μg/m^3	Per 0.1 μg/m^3	Per IQR μg/m^3	Per 90IPR μg/m^3
Smoke $PM_{2.5}$	1.032 (1.027-1.037)	1.003 (1.003-1.004)	1.010 (1.008-1.011)	1.029 (1.025-1.034)
Smoke carbon	1.087 (1.076-1.098)	1.008 (1.007-1.009)	1.021 (1.018-1.023)	1.055 (1.048-1.062)
Non-smoke $PM_{2.5}$	1.009 (1.008-1.011)	1.001 (1.001-1.001)	1.029 (1.025-1.033)	1.073 (1.063-1.083)
Non-smoke carbon	1.013 (1.010-1.017)	1.001 (1.001-1.002)	1.012 (1.009-1.016)	1.035 (1.025-1.045)

Table S8. Sensitivity analyses results.

**Sensitivity analyses**	**Hazard Ratio per 1 μg/m^3 (95% CI)**	
	**Smoke $PM_{2.5}$ mass**	**Smoke Carbon**
**Main results**	**1.032 (1.027-1.037)**	**1.087 (1.076-1.098)**
1-year moving average	1.012 (1.010-1.015)	1.062 (1.053-1.070)
2-year moving average	1.023 (1.019-1.027)	1.077 (1.067-1.087)
5-year moving average	1.040 (1.034-1.046)	1.093 (1.081-1.107)
Non-mover	1.023 (1.017-1.029)	1.073 (1.060-1.086)
Non-movers in exposure-specific low-variability ZIP codes	1.019 (1.009-1.031)	1.072 (1.047-1.098)
Exclude temperature and humidity	1.041 (1.035-1.046)	1.101 (1.089-1.113)
Combine smoke regime 1 and 2	1.026 (1.021-1.031)	1.078 (1.069-1.090)
3-year moving average Smoke OC	Smoke: 1.102 (1.089-1.116); Non-smoke 1.013 (1.008-1.018)	
3-year moving average Smoke EC	Smoke: 1.502 (1.422-1.587); Non-smoke 1.059 (1.048-1.070)	
3-year moving average Smoke non-carbon	Smoke: 1.010 (1.004-1.017); Non-smoke 1.012 (1.010-1.013)	
3-year moving average OC and EC adjustment	Smoke OC: 1.108 (1.084-1.132); Smoke EC: 1.110 (1.007-1.213)	
3-year moving average OC, EC, and non-carbon adjustment	Smoke OC: 1.143 (1.118-1.169); Smoke EC: 1.140 (1.033-1.259)	
3-year unweighted exposure	1.073 (1.063-1.084)	1.121 (1.107-1.135)
Additional adjustment for claims-based tobacco, alcohol, and drug use disorders	1.028 (1.023-1.033)	1.071 (1.061-1.081)

Table S9. Annual attributable deaths for non-smoke and smoke $PM_{2.5}$ among U.S. older adults (2005-2019), with allocation to carbonaceous and non-carbonaceous fraction.

Year	Non-smoke $PM_{2.5}$	Smoke $PM_{2.5}$	Smoke Carbon	Smoke Non-carbon
2005	122705.0 (97010.6-152134.3)	14519.2 (12281.8-16788.3)	13414.1 (11218.7-15731.3)	1105.1 (451.8-1773.8)
2006	120884.7 (95601.6-149857.0)	14529.6 (12290.5-16800.1)	13747.3 (11554.1-16025.0)	782.3 (308.7-1281.9)
2007	117580.1 (92663.4-146315.1)	16745.3 (14166.9-19359.0)	15741.4 (13223.5-18360.6)	1003.9 (394.7-1648.0)
2008	119807.0 (94479.7-149400.9)	22335.2 (18902.4-25813.0)	20688.8 (17345.3-24189.9)	1646.4 (652.3-2688.8)
2009	109606.5 (85466.3-138016.6)	25055.6 (21210.9-28949.1)	22856.4 (19105.1-26820.5)	2199.2 (886.4-3555.6)
2010	104445.1 (80335.6-132816.4)	25688.8 (21745.3-29684.1)	23310.6 (19460.7-27396.1)	2378.1 (965.2-3830.2)
2011	98940.2 (74808.8-127144.5)	23014.8 (19476.7-26602.1)	21084.3 (17627.1-24736.4)	1930.5 (783.2-3112.4)
2012	97260.7 (72975.0-125599.8)	22809.1 (19301.2-26366.7)	21348.1 (17920.4-24926.7)	1461.1 (581.0-2383.6)
2013	97444.9 (72689.2-126295.6)	23410.0 (19811.7-27059.0)	22201.5 (18672.6-25865.0)	1208.4 (479.4-1975.3)
2014	93591.6 (68909.8-122331.2)	24336.0 (20597.6-28127.7)	23039.6 (19368.8-26854.1)	1296.4 (517.5-2110.9)
2015	91076.9 (65779.9-120363.9)	23466.6 (19860.8-27124.3)	22172.0 (18633.1-25852.9)	1294.7 (517.0-2107.0)
2016	87424.9 (62220.2-116567.9)	23525.3 (19910.5-27192.1)	22137.3 (18591.8-25833.2)	1388.0 (555.6-2255.3)
2017	83933.7 (58274.8-113568.2)	25276.4 (21391.6-29217.3)	23876.8 (20066.6-27836.6)	1399.6 (557.1-2282.6)
2018	79206.1 (53599.1-109014.0)	33455.8 (28327.9-38653.0)	31145.1 (26103.6-36437.0)	2310.7 (943.1-3711.9)
2019	76508.5 (50962.2-106465.5)	40554.0 (34357.1-46828.4)	37262.5 (31146.0-43750.2)	3291.6 (1373.3-5218.3)
Average	93776.0 (70361.0-120993.2)	22420.1 (18977.0-25910.3)	20876.6 (17502.4-24413.5)	1543.5 (622.9-2496.0)

Table S10. State-level annual attributable mortality rates (per 100,000) and attributable deaths for non-smoke and smoke $PM_{2.5}$ among U.S. older adults (2005-2019), with allocation to carbonaceous and non-carbonaceous fraction.

State	Smoke $PM_{2.5}$ Mortality Rate[1]	Non-smoke $PM_{2.5}$	Smoke $PM_{2.5}$	Smoke Carbon	Smoke Non-carbon
Montana	226.9 (192.8-261.3)	133.1 (53.4-222.1)	368.2 (312.8-424.0)	316.6 (261.9-376.4)	51.6 (21.5-81.5)
Idaho	166.3 (141.1-191.7)	316.3 (202.7-442.4)	363.9 (308.8-419.5)	315.7 (261.6-374.1)	48.2 (19.9-76.7)
Wyoming	124.8 (105.8-144.0)	50.2 (16.4-90.2)	96.7 (82.0-111.6)	87.3 (72.9-102.6)	9.4 (3.8-15.0)
Georgia	109.9 (93.1-126.9)	3000.9 (2297.0-3783.5)	1277.1 (1081.6-1475.0)	1223.6 (1032.0-1421.0)	53.4 (20.7-88.5)
Alabama	107.9 (91.4-124.7)	2092.3 (1607.0-2631.4)	766.2 (648.7-885.1)	728.8 (614.2-847.1)	37.4 (14.4-62.2)
Oregon	104.8 (88.9-121.0)	847.9 (534.1-1196.4)	625.5 (530.3-721.7)	555.3 (461.9-655.9)	70.1 (29.1-111.4)
Arkansas	102.3 (86.6-118.3)	1225.5 (931.7-1553.0)	457.8 (387.6-528.9)	437.4 (368.7-508.5)	20.3 (7.9-33.6)
Mississippi	99.3 (84.0-114.7)	1135.8 (858.0-1445.4)	407.2 (344.7-470.5)	385.7 (324.5-449.0)	21.5 (8.4-35.4)
South Dakota	97.3 (82.5-112.4)	153.1 (85.1-228.1)	122.5 (103.8-141.5)	111.6 (93.3-130.8)	11.0 (4.4-17.8)
South Carolina	88.8 (75.2-102.6)	1728.8 (1309.2-2195.6)	632.0 (535.1-730.3)	602.1 (507.4-699.8)	29.9 (11.5-49.7)
Utah	86.6 (73.3-100.1)	422.2 (280.9-579.7)	241.3 (204.3-278.8)	216.8 (180.8-255.1)	24.5 (9.9-39.5)
Kansas	85.2 (72.1-98.5)	880.3 (629.1-1160.1)	343.4 (290.6-396.9)	322.0 (270.6-375.4)	21.4 (8.4-35.3)
North Carolina	84.5 (71.5-97.6)	3188.8 (2381.6-4086.4)	1157.5 (980.2-1337.1)	1078.4 (904.0-1261.4)	79.1 (32.3-127.0)
Oklahoma	83.9 (71.0-97.0)	1357.6 (1003.6-1752.7)	453.5 (383.8-524.3)	433.9 (365.7-504.1)	19.7 (7.6-32.8)
Louisiana	83.1 (70.3-96.0)	1605.0 (1212.9-2042.3)	505.3 (427.7-583.9)	475.6 (399.9-554.2)	29.7 (11.6-48.9)
Nebraska	80.1 (67.8-92.6)	498.0 (341.4-672.0)	210.8 (178.4-243.6)	191.5 (160.0-224.8)	19.3 (7.7-31.3)
North Dakota	78.4 (66.4-90.6)	125.6 (68.7-188.2)	80.7 (68.3-93.3)	75.2 (63.1-87.7)	5.6 (2.2-9.2)
Washington	78.3 (66.4-90.4)	1088.4 (626.5-1599.0)	727.7 (616.6-840.2)	659.0 (550.2-774.5)	68.7 (28.0-110.3)
Colorado	77.7 (65.8-89.8)	593.2 (307.8-909.2)	488.6 (413.9-564.4)	440.9 (367.5-519.4)	47.7 (19.8-75.8)
West Virginia	76.4 (64.6-88.2)	887.5 (669.9-1129.3)	242.1 (205.0-279.8)	227.0 (190.5-265.3)	15.1 (6.2-24.2)
Tennessee	70.9 (60.0-82.0)	2619.3 (2007.7-3298.8)	659.8 (558.4-762.6)	636.5 (536.7-739.3)	23.3 (9.1-38.4)
Missouri	61.6 (52.1-71.3)	2631.9 (2036.6-3293.2)	552.5 (467.4-638.7)	532.4 (448.9-618.3)	20.0 (7.7-33.4)
Iowa	58.6 (49.5-67.7)	1132.3 (831.4-1468.1)	281.3 (237.9-325.2)	264.8 (222.7-308.6)	16.5 (6.4-27.2)
Kentucky	57.7 (48.8-66.8)	1934.1 (1496.8-2419.1)	360.9 (305.3-417.3)	347.0 (292.6-403.1)	13.8 (5.3-23.1)
New Mexico	55.7 (47.2-64.4)	277.5 (141.6-429.8)	167.2 (141.5-193.2)	156.5 (131.3-182.9)	10.7 (4.3-17.3)
Virginia	54.8 (46.4-63.4)	2440.5 (1823.8-3126.2)	592.2 (501.1-684.5)	559.2 (469.8-652.4)	32.9 (13.2-53.5)
California	54.7 (46.3-63.1)	11611.6 (9016.9-14484.7)	2567.2 (2174.0-2965.5)	2209.1 (1821.3-2635.0)	358.2 (151.2-563.2)
Nevada	51.3 (43.4-59.3)	532.1 (349.4-735.2)	190.3 (161.1-219.9)	180.8 (152.3-210.3)	9.5 (3.7-15.7)
Texas	50.8 (43.0-58.8)	6555.6 (4909.3-8392.5)	1476.5 (1249.2-1707.1)	1446.0 (1221.2-1675.9)	30.5 (11.7-50.9)

Minnesota	50.1 (42.4-57.9)	1468.8 (1055.8-1928.6)	373.4 (315.9-431.8)	358.6 (302.3-416.6)	14.9 (5.7-24.8)
Wisconsin	48.4 (40.9-55.9)	1807.1 (1308.5-2362.8)	407.2 (344.4-470.9)	382.2 (321.0-445.9)	25.0 (9.8-41.1)
Indiana	44.0 (37.2-50.8)	2871.0 (2244.4-3566.1)	398.0 (336.6-460.3)	370.7 (310.9-433.1)	27.3 (10.7-44.7)
Florida	43.4 (36.7-50.2)	5836.8 (4082.8-7792.9)	1564.1 (1323.2-1808.6)	1475.8 (1240.2-1720.6)	88.3 (34.8-144.9)
Pennsylvania	39.6 (33.5-45.8)	6163.6 (4757.2-7724.8)	822.3 (695.5-951.1)	769.9 (646.1-899.0)	52.4 (20.7-85.9)
Washington D.C.	36.8 (31.1-42.6)	215.8 (170.0-266.7)	27.1 (22.9-31.4)	24.6 (20.5-28.9)	2.5 (1.0-4.1)
Ohio	36.3 (30.7-42.0)	5488.0 (4280.1-6828.3)	630.3 (533.0-729.1)	598.7 (503.7-697.0)	31.7 (12.3-52.5)
Vermont	35.9 (30.4-41.6)	128.2 (76.1-185.9)	36.0 (30.5-41.7)	33.1 (27.7-38.8)	2.9 (1.2-4.7)
Illinois	35.4 (29.9-40.9)	5496.7 (4352.3-6763.7)	610.6 (516.4-706.2)	569.0 (477.3-664.9)	41.6 (16.4-68.3)
Maryland	34.3 (29.0-39.7)	2055.2 (1591.2-2570.6)	266.5 (225.4-308.3)	251.3 (211.0-293.3)	15.2 (6.0-24.9)
Delaware	34.3 (29.0-39.6)	315.9 (235.9-405.0)	49.6 (42.0-57.4)	45.2 (37.8-53.1)	4.4 (1.8-7.2)
Michigan	32.2 (27.2-37.2)	3635.3 (2719.5-4653.7)	472.6 (399.6-546.6)	450.2 (378.7-524.2)	22.3 (8.7-36.8)
New Hampshire	31.2 (26.4-36.1)	268.8 (164.6-384.2)	62.1 (52.5-71.9)	58.7 (49.4-68.4)	3.4 (1.3-5.6)
Maine	28.6 (24.1-33.0)	269.9 (145.2-407.7)	65.9 (55.7-76.3)	60.7 (50.8-71.1)	5.2 (2.1-8.5)
New Jersey	28.4 (24.0-32.8)	3020.9 (2275.0-3850.9)	359.2 (303.7-415.5)	333.5 (279.4-390.3)	25.6 (10.2-41.8)
Rhode Island	26.4 (22.3-30.5)	302.3 (205.3-410.3)	42.8 (36.1-49.5)	40.5 (34.0-47.2)	2.3 (0.9-3.8)
Arizona	26.1 (22.1-30.1)	1361.0 (900.2-1872.9)	260.0 (220.0-300.6)	242.1 (202.9-283.1)	17.9 (7.2-29.1)
Massachusetts	24.5 (20.7-28.4)	1816.1 (1266.0-2429.1)	239.7 (202.6-277.3)	228.2 (191.9-265.9)	11.5 (4.5-18.8)
New York	24.4 (20.6-28.2)	6083.0 (4528.6-7810.8)	685.0 (579.1-792.4)	636.3 (532.9-744.6)	48.7 (19.5-79.2)
Connecticut	23.4 (19.7-27.0)	959.2 (662.7-1289.6)	125.7 (106.3-145.5)	121.7 (102.6-141.4)	4.0 (1.5-6.7)

[1]Mortality rate attributable to smoke $PM_{2.5}$ (per 100,000 people per year).

Supplementary Figures

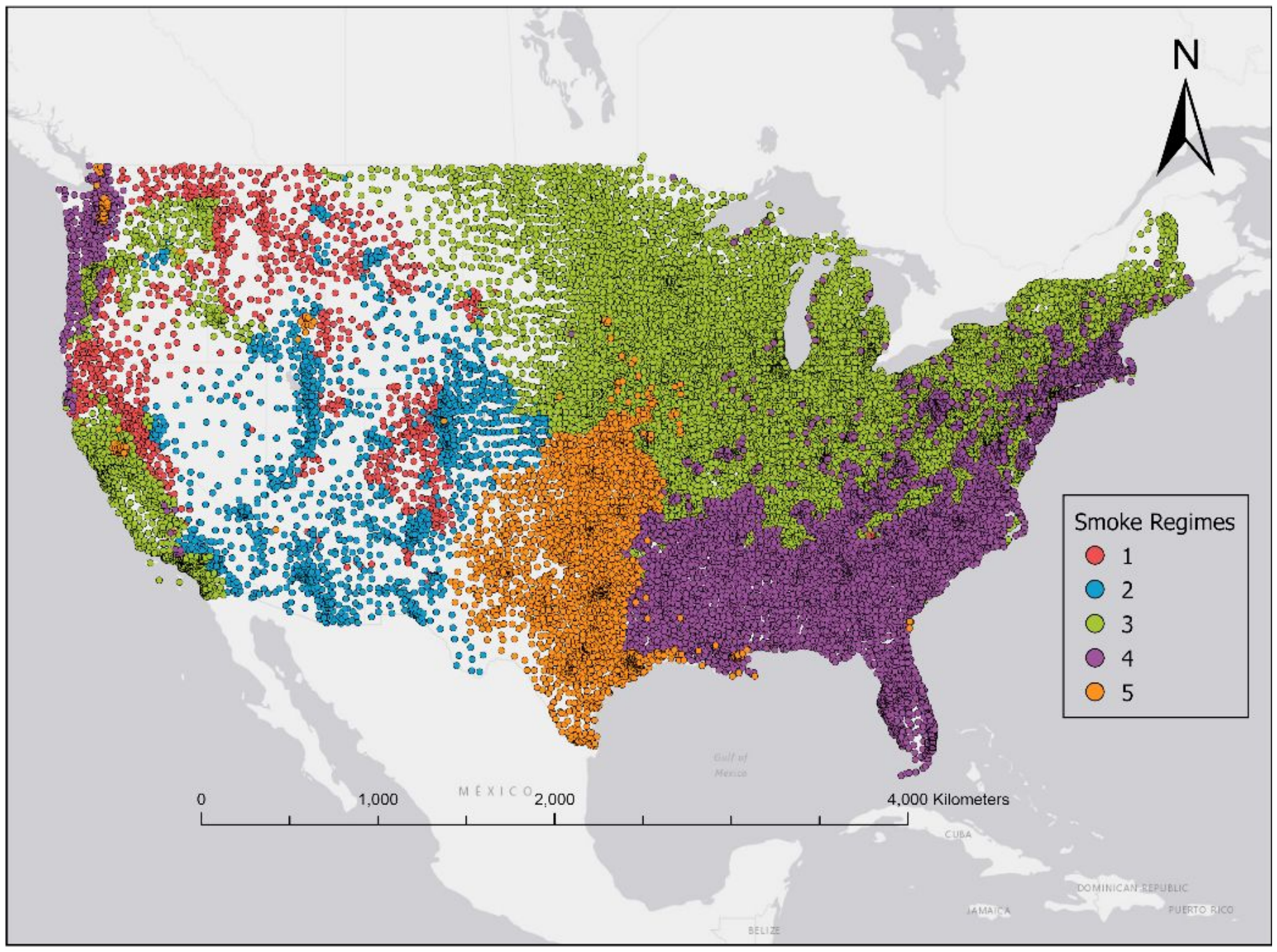

Figure S1. Five smoke regimes by ZIP codes. ZIP codes with the same color belong to the same smoke regime. The points denote the Zip code centroids.

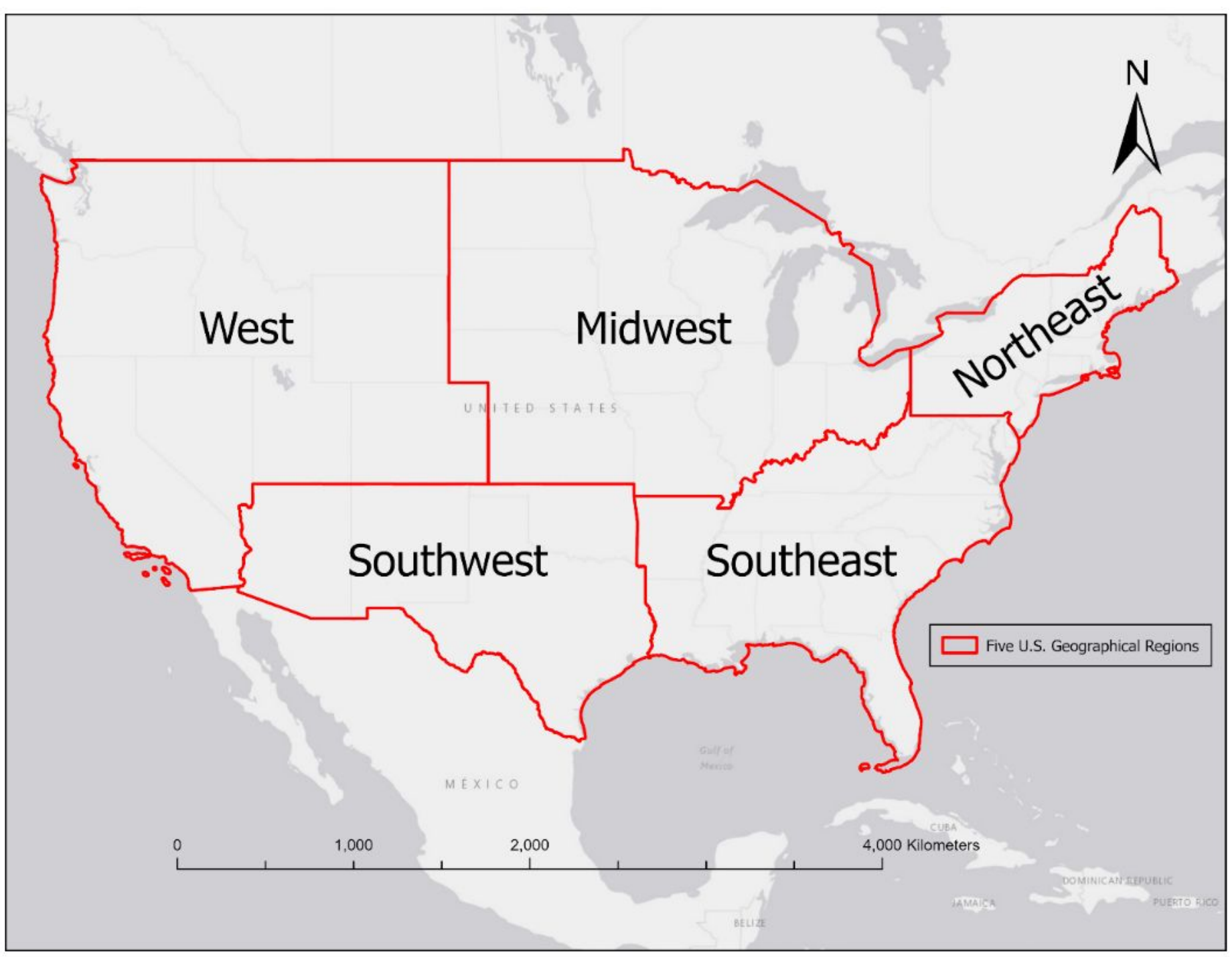

Figure S2. Five U.S. geographical regions used in this study (Northeast, Southeast, Midwest, West, Southwest).

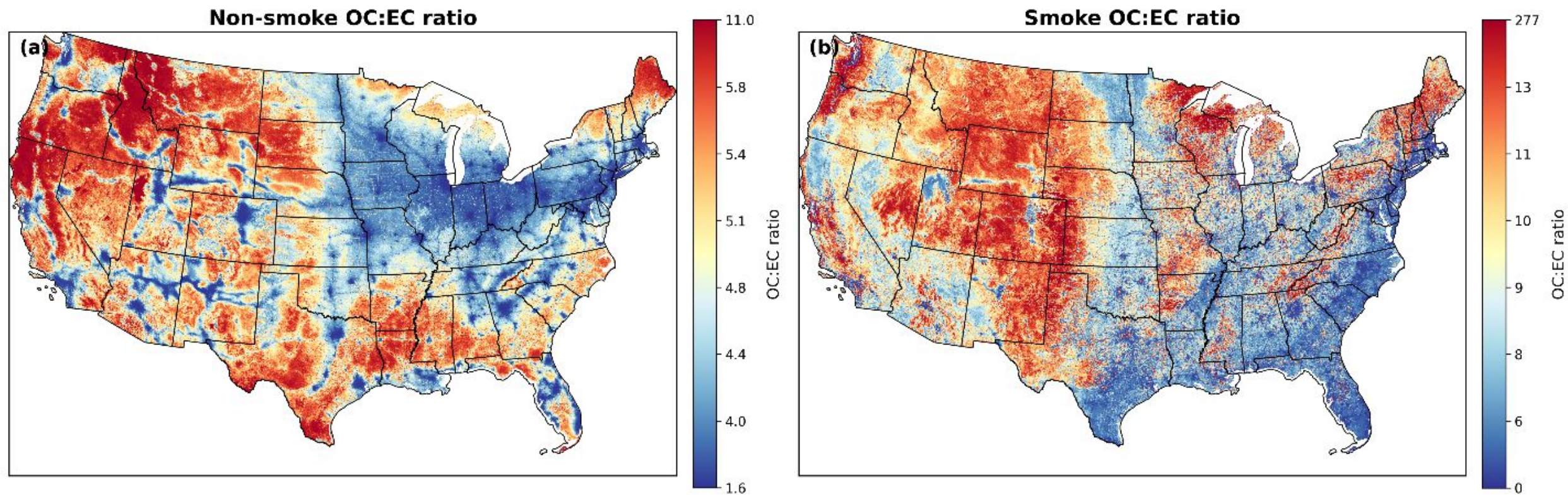

Figure S3. Mean non-smoke (left) and smoke (right) OC-to-EC ratio across the CONUS, 2002-2019. Mapped with 1-km spatial resolution.

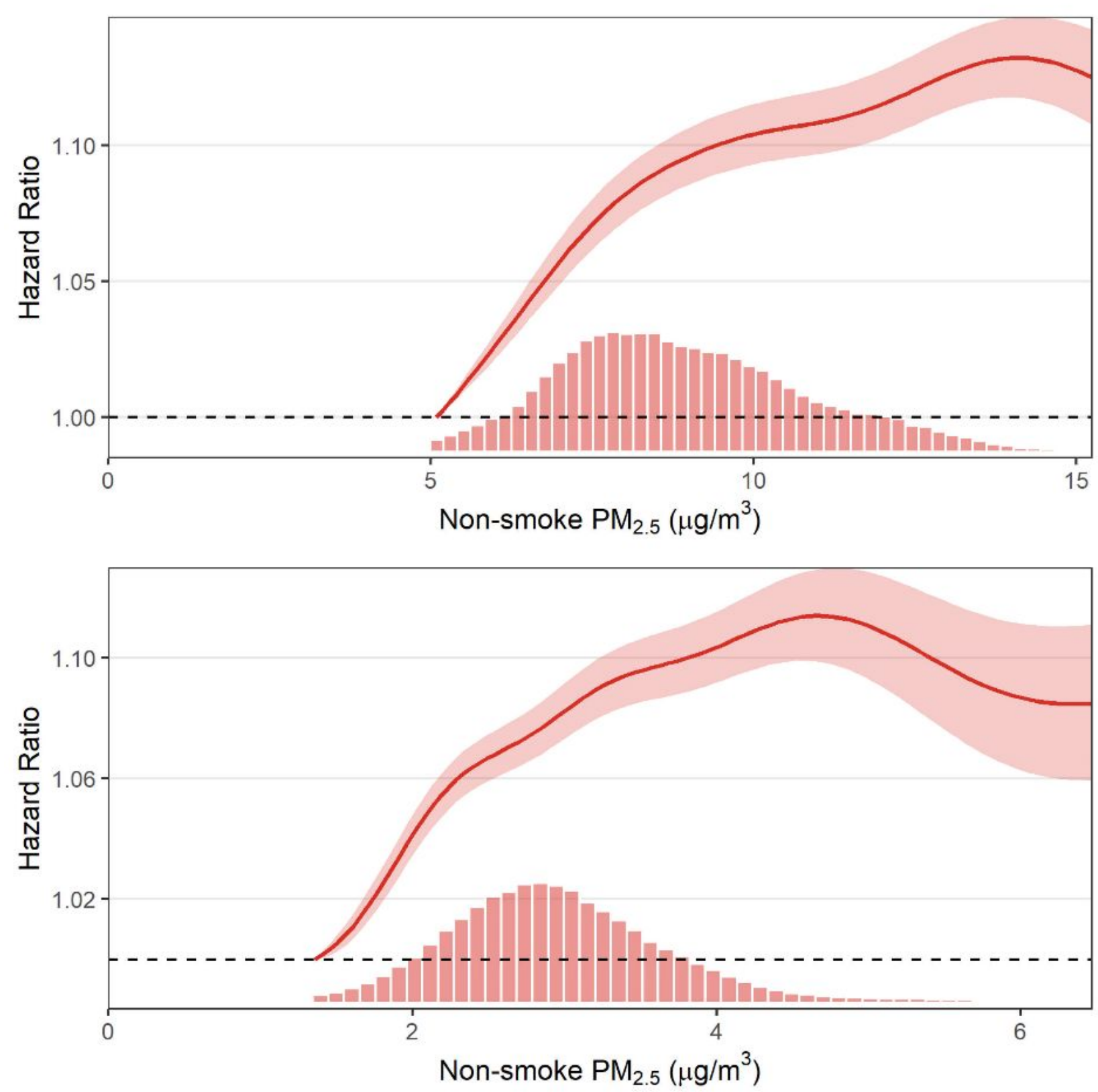

Figure S4. Exposure-response functions for 3-year moving-average concentrations of non-smoke $PM_{2.5}$ mass and non-smoke carbon in relation to all-cause mortality. The concentrations span the 1st to the 99th percentiles for each pollutant.

References

(1) Zanobetti, A.; Austin, E.; Coull, B. A.; Schwartz, J.; Koutrakis, P. Health effects of multi-pollutant profiles. *Environ Int* **2014**, *71*, 13–19. DOI: 10.1016/j.envint.2014.05.023 From NLM.

Di, Q.; Wang, Y.; Zanobetti, A.; Wang, Y.; Koutrakis, P.; Choirat, C.; Dominici, F.; Schwartz, J. D. Air pollution and mortality in the Medicare population. *New England Journal of Medicine* **2017**, *376* (26), 2513–2522.
(2) Winkler, R. L.; Mockrin, M. H. *Aging and Wildfire Risk to Communities*; U.S. Department of Agriculture, Economic Research Service, Washington, DC, 2025. https://research.fs.usda.gov/treesearch/69056DOI: 10.22004/ag.econ.352131.
(3) Eide, G. E. Attributable fractions for partitioning risk and evaluating disease prevention: a practical guide. *The clinical respiratory journal* **2008**, *2*, 92–103. Gefeller, O.; Land, M.; Eide, G. E. Averaging attributable fractions in the multifactorial situation: assumptions and interpretation. *Journal of Clinical Epidemiology* **1998**, *51* (5), 437–441.
(4) Shorrocks, A. F. Decomposition procedures for distributional analysis: a unified framework based on the Shapley value. *Journal of Economic Inequality* **2013**, *11* (1), 99–126.
(5) Murray, C. J.; Aravkin, A. Y.; Zheng, P.; Abbafati, C.; Abbas, K. M.; Abbasi-Kangevari, M.; Abd-Allah, F.; Abdelalim, A.; Abdollahi, M.; Abdollahpour, I. Global burden of 87 risk factors in 204 countries and territories, 1990–2019: a systematic analysis for the Global Burden of Disease Study 2019. *The Lancet* **2020**, *396* (10258), 1223–1249. Burnett, R. T.; Pope III, C. A.; Ezzati, M.; Olives, C.; Lim, S. S.; Mehta, S.; Shin, H. H.; Singh, G.; Hubbell, B.; Brauer, M. An integrated risk function for estimating the global burden of disease attributable to ambient fine particulate matter exposure. *Environmental health perspectives* **2014**, *122* (4), 397–403.
